# Diversity and Pathogenicity of *Fusarium* Root Rot Fungi from Canola (*Brassica napus*) in Alberta, Canada

**DOI:** 10.3390/ijms25116244

**Published:** 2024-06-05

**Authors:** Haitian Yu, Kan-Fa Chang, Rudolph Fredua-Agyeman, Sheau-Fang Hwang, Stephen E. Strelkov

**Affiliations:** 1 Department of Agricultural, Food and Nutritional Science, University of Alberta, Edmonton, AB T6G 2P5, Canada; haitian7@ualberta.ca (H.Y.); kanfa@ualberta.ca (K.-F.C.); freduaag@ualberta.ca (R.F.-A.); 2 Institute of Food Crops, Yunnan Academy of Agricultural Science, Kunming 650205, China

**Keywords:** identification, pathogenicity, diversity, root rot, canola, *Fusarium* spp.

## Abstract

Root rot disease poses a significant threat to canola (*Brassica napus*), underscoring the need for a comprehensive understanding of its causal agents for more effective disease mitigation. The composition and diversity of fungal pathogens associated with root rot of canola in Alberta, Canada, were evaluated from plant tissue samples collected in 2021 and 2022. The study revealed *Fusarium* spp. as the predominant pathogens found in almost all surveyed fields. *Fusarium avenaceum*, *F. redolens*, and *F. solani* were among the most frequently recovered species. Greenhouse trials confirmed their pathogenicity, with *F. avenaceum* and *F. sporotrichioides* found to be particularly aggressive. Additionally, *F. sporotrichioides* and *F. commune* were identified for the first time as canola root rot pathogens. Inoculation with isolates of most species resulted in significant reductions in seedling emergence, plant height, and shoot and root dry weights. Analysis of translation elongation factor 1-α (TEF-1α) and internal transcribed spacer (ITS) sequences confirmed the identity of the *Fusarium* spp., while concatenating the ITS and TEF-1α sequences enabled improved species differentiation. Geographic and year effects did not influence fungal diversity or aggressiveness, as determined by principal component analysis. This study emphasized the high diversity and impact of *Fusarium* spp. in causing canola root rot.

## 1. Introduction

Root rot is a widely distributed disease causing significant yield losses in many agriculturally important crops worldwide [1]. The pathogens responsible for this disease are collectively referred to as the root rot complex (RRC), which consists mostly of fungi and fungal-like microorganisms (oomycetes) and, to a lesser extent, some bacteria and viruses [1]. The first indicators of root rot include poor germination and reduced emergence [2,3]. Disease development can also be accompanied by aboveground symptoms such as crown rot [4], leaf yellowing [5], wilting, and stunting [6]. Plants typically cannot recover once these aboveground symptoms appear [7], and the severity of root rot is exacerbated under wet conditions. In Alberta and other provinces of western Canada, root rot represents an important constraint to canola (syn. oilseed rape; *Brassica napus* L.) production, causing yield losses of up to 35% [8]. This is a cause for concern, as canola contributes CAD 29.9 billion annually to the national economy [9], with the vast majority of production in western Canada.

Root rot first emerged as a threat to Canadian canola production in the late 1980s, approximately 10 years after the start of commercial cultivation of this crop [10,11,12]. In studies conducted in the 1980s and 1990s, the primary cause appeared to be *Rhizoctonia solani* J.G. Kühn [10,11,12], although *Fusarium avenaceum* R.J. Cook was also reported as one of the causal agents of canola seedling blight during this period [13]. By the 2000s, *Fusarium* spp. had emerged as a major component of the canola RRC [14,15,16] together with *R. solani* [15,16,17,18,19]. Surveys conducted in central Alberta in the early 2000s identified *Fusarium acuminatum* Ellis & Everh as the predominant *Fusarium* species and *F. avenaceum* as the most aggressive species causing seedling blight of canola [14]. However, the last major analysis of *Fusarium* spp. on canola in Alberta was conducted on samples collected from 2009 to 2011 [14]. As such, information on pathogen composition in this region is somewhat outdated, particularly given that numerous other *Fusarium* spp. have been implicated in root rot of canola and other *Brassicas*.

In Australia, *F. acuminatum* and *Fusarium semitectum* (Desm.) Sacc. were identified as the major agents causing root diseases in canola seedlings and adult plants [20]. In the United States, *F. acuminatum*, *Fusarium oxysporum* Schlecht. emend. Snyder & Hansen, *Fusarium solani* (Mart.) Sacc, and *Fusarium sporotrichioides* Sherb. were recovered from diseased root samples of *Brassica carinata* L., and the virulence of these species was confirmed on several cultivars of this crop [21]. In Poland, foot rot of rapeseed seedlings caused by *F. oxysporum* and *Fusarium culmorum* (Wm.G.Sm.) Sacc. is commonly encountered [22]. A field survey in Iran identified *F. solani* as the predominant species causing root and crown rot in canola, while *Fusarium equiseti* (Corda) Sacc. was the most aggressive [23,24]; recent studies also isolated *F. acuminatum* and *Fusarium clavum* J.W. Xia, L. Lombard, Sand.-Den., X.G. Zhang & Crous from diseased canola [25]. On vegetable *Brassicas*, most studies have concentrated on Fusarium wilt and its control [26,27,28]. However, root rot, despite posing a significant threat to *Brassica* production, has received comparatively little attention.

With over 80 species worldwide, a wide host range, and rapid growth, *Fusarium* spp. have become important constraints to the production of canola and other crops [29]. The identification of individual species of *Fusarium* has traditionally relied on colony and morphological characteristics [30,31]. More recently, species identification and understanding of *Fusarium* taxonomy have been enhanced by the advent of modern genetic, PCR-based, and sequencing technologies. Compared with traditional methods based on morphological characteristics, molecular analysis of conserved gene regions, such as the internal transcribed spacer (ITS) region, the translation elongation factor 1-α (TEF-1α) gene, the intergenic spacer (IGS) region, and RNA polymerase II (RPB2) gene enables more precise species identification and improved knowledge of species diversity and distribution [32,33,34,35,36].

The management of root rot can be challenging. Only partial resistance, controlled by numerous quantitative trait loci, has been reported in canola [37]. Cultural practices and fungicidal seed treatments can improve canola emergence and yield, but not necessarily under high disease pressure [38]. Other strategies, such as diverse crop rotations [16,39], have also been suggested to reduce root rot severity. However, none of these strategies are sufficient to combat root rot, and an integrated approach is required. This study aims to characterize *Fusarium* spp. associated with canola root rot in Alberta, providing the first comprehensive data on this subject in over a decade. The findings highlight the high prevalence, pathogenicity, and diversity of root rot pathogens affecting canola crops in this province. This information can serve as a foundational resource for the development of integrated management strategies, which can enhance the resilience of canola plants against root rot and contribute to more effective agricultural practices.

## 2. Results

### 2.1. Root Rot Incidence and Pathogen Recovery

**Figure 1:**
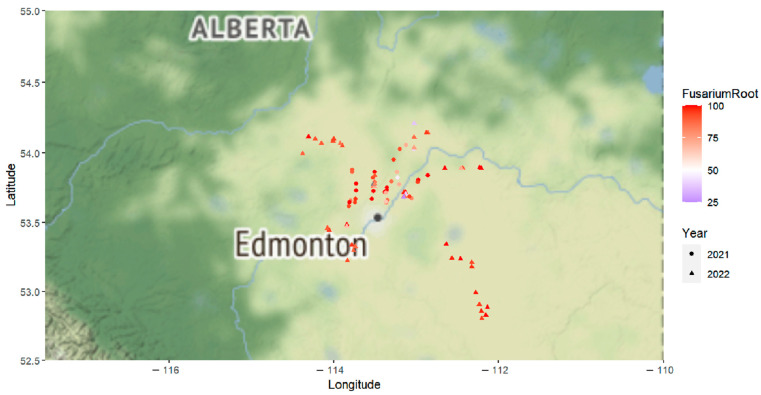
The incidence of *Fusarium* recovered from diseased canola root samples collected across 86 field sites in 2021 and 2022. Longitude and latitude indicate the position of surveyed fields on the maps. Fields visited in 2021 and 2022 are indicated as circles and triangles. “FusariumRoot” represents the incidence (%) of *Fusarium* spp. recovered from root samples within each surveyed field, as reflected on the color scale.

In 2021, root rot was identified in 34 of the 35 surveyed fields, with disease incidence ranging from 0% to 50% and averaging 1.7% [40]. In the lab, *Fusarium* spp. were recovered from 41.7% to 100% of the diseased root samples collected from each field and an average of 85.0% across all fields (Figure 1 and Figure S1, and Table S1). While other fungi, including *R. solani*, were also recovered from the root samples, their incidence was relatively low. *Fusarium* was also the predominant genus recovered from diseased lower stem samples collected in 2021.

Root rot was detected in all of the 51 fields surveyed in 2022, with the disease incidence (average of 19.6% and range of 1% to 51%) generally much higher than in 2021 [41]. As in 2021, *Fusarium* spp. continued to be the most commonly found fungi, recovered from root samples collected from each field at rates of 25% to 100%. On average, *Fusarium* spp. were recovered from 88.2% of root samples across all fields (Figure 1 and Figure S1, and Table S1). *Rhizoctonia solani* and *Leptosphaeria maculans* (Sowerby) P. Karst were among the few other pathogens with recovery rates exceeding 10% in the root samples. Among the lower stem samples collected in 2022, *Fusarium* spp. were the most prevalent (recovered from 66.8% of samples), followed by *L. maculans* (present in >30% of samples).

### 2.2. Fusarium Species Identification and Prevalence

**Figure 2:**
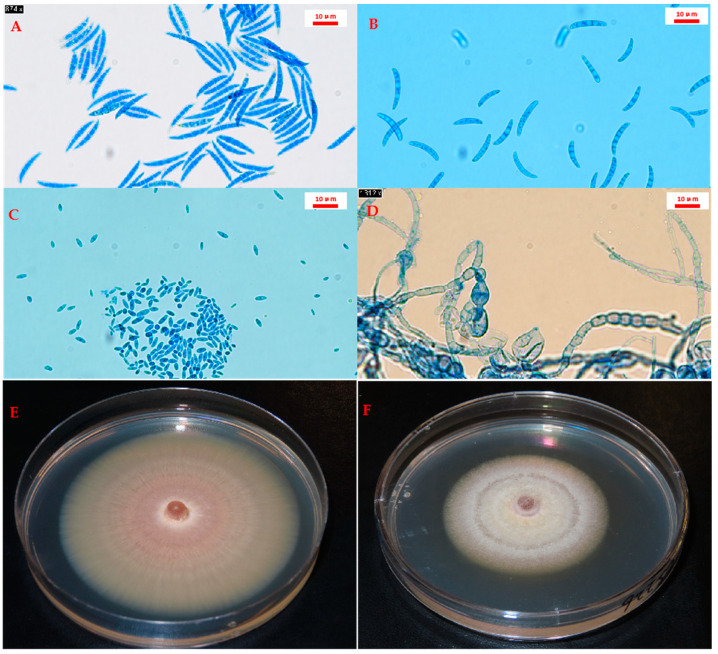
Microscopic structures of *Fusarium* formed on potato dextrose agar (PDA) medium. Examples show macroconidia of *Fusarium culmorum* (**A**) and *Fusarium solani* (**B**), microconidia of *Fusarium redolens* (**C**), and chlamydospores (**D**). Scale bars (10 µm) are indicated in red. Cultures of *F. solani* (**E**) and *F. redolens* (**F**) on PDA are shown in the bottom panels.

In 2021, a total of 207 purified isolates were recovered from 35 fields. One hundred fifty-one isolates from 26 fields were selected for species identification based on colony and morphological characteristics (Figure 2) combined with analysis of the TEF-1α gene and ITS region. Of these, 149 isolates from 26 fields were identified as *Fusarium*, representing 14 distinct species. These included *F. avenaceum* (74 isolates), *Fusarium redolens* Wollenw (18 isolates), *F. solani* (14 isolates), *Fusarium torulosum* (Berk. & M.A. Curtis) Nirenberg (11 isolates), *F. culmorum* (5 isolates), *F. equiseti* (4 isolates), *F. acuminatum* (3 isolates), *F. oxysporum* (2 isolates), *Fusarium tricinctum* (Corda) Sacc. (1 isolate), *Fusarium proliferatum* (Matsush.) Nirenberg ex Gerlach & Nirenberg (1 isolate), *Fusarium graminearum* (Schwein.) Petch (1 isolate), *Fusarium commune* K. Skovg., O’Donnell & Nirenberg (1 isolate), *F. sporotrichioides* (2 isolates), *Fusarium flocciferum* Corda (1 isolate), and an unidentified *Fusarium* species (Table S1). *Fusarium avenaceum* was the most prevalent species, found in 20 of the 26 fields and representing 49.7% of all *Fusarium* isolates. The next most common species included *F. redolens* (12.1%), *F. solani* (9.4%), and *F. torulosum* (7.4%).

In 2022, a total of 427 purified isolates were recovered from 51 fields. A total of 79 isolates from 32 fields were selected for species identification, with 75 of these identified as *Fusarium*, representing nine species. These included *F. avenaceum* (36 isolates), *F. solani* (8 isolates), *F. redolens* (7 isolates), *F. oxysporum* (7 isolates), *F. culmorum* (5 isolates), *F. acuminatum* (4 isolates), *F. torulosum* (2 isolates), *F. tricinctum* (2 isolates), *F. flocciferum* (1 isolate), and 3 isolates of an unidentified *Fusarium* species (Table S1). As of 2021, *F. avenaceum* was the most commonly identified species, confirmed in 22 out of 32 fields and representing 48% of all *Fusarium* isolates. The next most common species found in 2022 was *F. solani* (10.7%), alongside *F. redolens* and *F. oxysporum* (both 9.3%). Three species—*F. commune*, *F. equiseti*, and *F. sporotrichioides*—were identified only in the collections made in 2021.

### 2.3. Pathogenicity Test

To evaluate the pathogenicity of various *Fusarium* isolates on canola, multiple parameters, including disease severity, seedling emergence, and shoot and root dry weights, were assessed at the seedling stage across two repeated experiments. Analysis of variance (ANOVA) indicated no significant (*p* > 0.05) variation in disease severity or other growth parameters (emergence and root and shoot dry weights) between the two repeats of the greenhouse trial, suggesting the consistency of results between the repeats. Therefore, the data were combined for further analysis. In contrast, significant variation (*p* < 0.05) among treatments (isolates) was observed in both years. The results of a *t*-test comparing the inoculated treatments vs. non-inoculated control for all parameters in each respective year are presented in Supplementary Figures S2 and S3. As expected, in both years, all the isolates exhibited a significant increase in disease severity relative to the non-inoculated control (Figures S2A and S3A). Out of the 130 isolates tested from the 2021 survey, 98 caused a reduction in canola seedling emergence (i.e., pre-emergence damping-off) (Figure S2B) and shoot dry weight (Figure S2D), while 82 isolates caused a decrease in plant height (Figure S2C) and 126 isolates caused a decrease in root dry weight (Figure S2E). Among the 75 isolates tested from the 2022 survey, 49 reduced seedling emergence (Figure S3B), 52 reduced plant height (Figure S3C), and 49 and 74 reduced shoot (Figure S3D) and root (Figure S3E) weight, respectively.

#### 2.3.1. Effect of *Fusarium* spp. on Disease Severity

The disease severity data aggregated for all isolates collected in both years compared with the aggregated non-inoculated controls are shown in Table S1. Average disease severity ranged from 0.58 to 4.0 (Table 1). Three isolates, each of *F. solani* and *F. redolens,* induced disease severities of <1.0 (Table 1). Eighty-three isolates caused intermediate symptoms, with disease severities ranging from 1.0 to 2.0. These included 21 isolates of *F. redolens*, 18 of *F. avenaceum*, 16 of *F. solani*, and 10 isolates of *F. torulosum*, as well as between 1 and 5 isolates each of *F. culmorum*, *F. oxysporum*, *F. acuminatum*, *F. tricinctum*, *F. flocciferum*, *F. proliferatum*, and *F. equiseti* (Table 1). Seventy-six isolates caused disease severities ranging from 2.0 to 3.0. These represented 51 isolates of *F. avenaceum*, 5 of *F. culmorum*, 4 of *F. oxysporum*, and 2–3 isolates each of *F. acuminatum*, *F. equiseti*, *F. torulosum*, and *F. solani*, an unidentified *Fusarium* species, and 1 isolate each of *F. commune*, *F. graminearum*, and *F. redolens* (Table 1). Finally, 40 isolates caused disease severities > 3.0. Thirty-seven of these isolates were identified as *F. avenaceum*, two as *F. sporotrichioides*, and one could not be identified at the species level (Table 1).

#### 2.3.2. Effect of *Fusarium* spp. on Seedling Emergence

The reduction in seedling emergence caused by inoculation with *Fusarium* spp. ranged from 1.32% to 100% (Table 1). Seventy-four of the isolates representing 11 species caused a <25% reduction in emergence. These consisted of *F. redolens* (19 isolates), *F. solani* (18 isolates), *F. avenaceum* (11 isolates), *F. torulosum* (10 isolates), *F. acuminatum* (4 isolates), *F. culmorum* (4 isolates), *F. tricinctum* (3 isolates), *F. flocciferum* (2 isolates), *F. oxysporum* (1 isolate), *F. proliferatum* (1 isolate), and *F. commune* (1 isolate). Fifty-eight isolates representing nine *Fusarium* species caused a reduction in seedling emergence of 25% to 50%. These comprised 31 isolates of *F. avenaceum*, 5 isolates each of *F. redolens* and *F. culmorum*, 4 isolates each of *F. equiseti* and *F. oxysporum*, 3 isolates of *F. torulosum*, 2 isolates each of *F. solani* and *F. acuminatum*, 1 isolate each of *F. graminearum,* and an unidentified species. A total of 45 isolates caused reductions of 50% to 75% in seedling emergence, including 39 isolates of *Fusarium avenaceum* as the predominant species, 3 isolates of *F. oxysporum,* 1 isolate of *F. redolens,* and 2 unidentifiable isolates. Finally, 28 isolates reduced seedling emergence by >75% and consisted of 24 isolates of *F. avenaceum*, 2 isolates of *F. sporotrichioides,* and 1 isolate each of *F. culmorum* and *F. solani* (Table 1). Among all the isolates, only 58, including 17 isolates of *F. solani*, 14 isolates of *F. redolens*, 4 isolates of *F. culmorum*, 3 isolates each of *F. avenaceum*, *F. acuminatum*, *F. oxysporum*, and *F. tricinctum*, 1 isolate each of *F. commune*, *F. proliferatum*, *F. flocciferum*, and an unidentified species, did not significantly reduce seedling emergence (Figures S2B and S3B).

#### 2.3.3. Effect of *Fusarium* spp. on Plant Height

The effects of *Fusarium* spp. inoculation on plant height were mixed, with most isolates causing reductions in the range from 1.55% to 100% and a few causing small increases (Table 1). One-hundred and twenty-seven isolates, representing 12 different *Fusarium* species, were identified as causing plant height reductions of <25%. These included 66 isolates of *F. avenaceum*, 14 isolates of *F. redolens*, and 13 isolates of *F. solani*, as well as *F. torulosum* (8 isolates), *F. oxysporum* (7 isolates), *F. acuminatum* (6 isolates), *F. culmorum*, and *F. tricinctum* (3 isolates each). Additionally, there were two isolates each of *F. flocciferum* and an unidentified species, and one isolate each of *F. proliferatum*, *F. graminearum*, and *F. commune*, which caused reductions in plant height of <25%. Sixty of the tested isolates, representing seven species, caused intermediate reductions (25% to 50%) in plant height. These included 28 isolates of *F. avenaceum*, 11 isolates of *F. redolens*, 7 isolates of *F. solani,* 6 isolates each of *F. culmorum*, 5 isolates of *F. torulosum*, 1 isolate each of *F. equiseti*, *F. oxysporum*, and an unidentified species. A total of 16 isolates caused severe reductions (50% to 75%) in plant height, consisting of 11 isolates of *F. avenaceum*, 3 isolates of *F. equiseti,* and 1 isolate each of *F. solani* and *F. culmorum*. Two isolates of *F. sporotrichioides* caused very severe reductions in plant height of >75%. A total of 59 isolates, including 31 isolates each of *F. avenaceum*, 7 isolates of *F. solani*, 6 isolates of *F. torulosum*, 5 isolates of *F. redolens*, 2 isolates each of *F. culmorum*, *F. acuminatum*, *F. oxysporum*, and *F. flocciferum*, and one isolate of each of *F. tricinctum* and *F. graminearum* did not significantly reduce plant height (Figures S2B and S3B). Inoculation with 13 isolates representing seven species resulted in numerical increases in plant height, but these increases were significant (*p* < 0.05) only for 1 isolate each of *F. redolens* and *F. tricinctum* (Figures S2C and S3C).

#### 2.3.4. Effect of *Fusarium* spp. on Shoot Dry Weight

In terms of shoot dry weight, the reductions caused by inoculation with *Fusarium* spp. ranged from 0.06% to 98.6%, while a few isolates increased shoot dry weight by up to 67.9% (Table 2). Forty-nine of the isolates caused reductions of <25%. These included *F. avenaceum* (13 isolates), *F. solani* (10 isolates), *F. redolens* (10 isolates), *F. torulosum* (6 isolates), 2 isolates each of *F. acuminatum*, *F. flocciferum*, *F. tricinctum*, and *F. culmorum*, and 1 isolate each of *F. graminearum* and *F. oxysporum*. Sixty-seven isolates caused reductions in shoot dry weight of 25 to 50%. These represented nine species, including *F. avenaceum* (37 isolates), *F. redolens* (9 isolates), *F. oxysporum* (4 isolates), 3 isolates each of *F. torulosum*, *F. solani*, *F. acuminatum*, and *F. culmorum*, 2 isolates of *F. equiseti*, an unidentified species, and 1 isolate of *F. commune*. Twenty-eight isolates of *F. avenaceum*, three isolates of *F. solani*, two isolates of *F. equiseti*, and one isolate each of *F. redolens* and *F. culmorum* caused reductions in shoot dry weight of 50% to 75%. A total of 29 isolates, including *F. avenaceum* (25 isolates), *F. sporotrichioides* (2 isolates), *F. culmorum* (1 isolate), and an unidentified isolate induced reductions in shoot dry weight of >75% (Table 2). Among all the isolates, 12 isolates of *F. avenaceum*, 8 isolates of *F. redolens*, 5 isolates of *F. solani*, 2 isolates each of *F. culmorum* and *F. torulosum*, and 1 isolate each of *F. oxysporum*, *F. graminearum*, *F. tricinctum*, and *F. flocciferum* did not significantly reduce shoot dry weight (Figures S2D and S3D). In contrast, three isolates of *F. redolens*, two isolates each of *F. avenaceum* and *F. solani*, and one isolate each of *F. acuminatum*, *F. culmorum*, and *F. torulosum* induced significant (*p* < 0.05) increases in shoot dry weigh (Figures S2D and S3D).

#### 2.3.5. Effect of *Fusarium* spp. on Root Dry Weight

Reductions in root dry weight following inoculation with *Fusarium* spp. ranged from 11.9% to 99.6% (Table 2). Four isolates (two of *F. redolens*, one of *F. culmorum*, and one of *F. solani*) caused reductions of <25%. A total of 17 isolates representing seven species caused reductions in root dry weight of 25% to 50%, including 6 isolates of *F. avenaceum*, 3 isolates of *F. redolens*, 2 isolates each of *F. oxysporum*, *F. solani*, and *F. torulosum*, and 1 isolate each of *F. proliferatum* and *F. tricinctum*. Seventy-eight isolates, including 11 *Fusarium* species, caused reductions in root dry weight of 50% to 75%. These consisted of *F. avenaceum* (24 isolates), *F. redolens* (15 isolates), *F. solani* (11 isolates), *F. torulosum* (6 isolates), 5 isolates each of *F. acuminatum*, *F. oxysporum*, and *F. culmorum*, 2 isolates each of *F. tricinctum* and an unidentified species, and 1 isolate each of *F. commune*, *F. flocciferum*, and *F. graminearum*. The largest group of 106 isolates caused the most severe reductions (75% to 100%) in root dry weight and included *F. avenaceum* (75 isolates), *F. solani* (7 isolates), 5 isolates each of *F. redolens* and *F. torulosum*, 4 isolates each of *F. culmorum* and *F. equiseti*, 2 isolates of *F. sporotrichioides*, 1 isolate each of *F. acuminatum*, *F. flocciferum*, *F. oxysporum*, and an unidentified species (Table 2). Among all the isolates, only one *F. culmorum* isolate, CS250, did not significantly (*p* < 0.05) reduce the root rot of canola seedlings (Figure S3E).

#### 2.3.6. Comparison of Disease Severity between *Fusarium avenaceum* and other *Fusarium* Species

**Figure 3:**
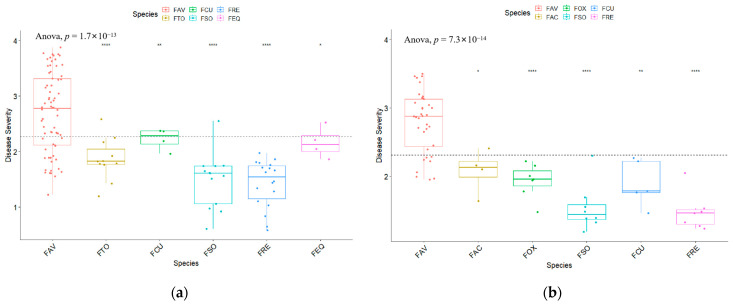
Root rot disease severity on the canola cv. ‘Westar’ caused by isolates representing different *Fusarium* spp. recovered in 2021 (**a**) and 2022 (**b**). FAV—*Fusarium avenaceum*; FCU—*Fusarium culmorum*; FRE—*Fusarium redolens*; FTO—*Fusarium torulosum*; FSO—*Fusarium solani*; FEQ—*Fusarium equiseti*; FOX—*Fusarium oxysporum*; and FAC—*Fusarium acuminatum*. Inoculations were conducted under greenhouse conditions and rated on a 0 to 4 scale [42], where 0 = healthy roots and 4 = tap root severely girdled, brown lesions on >75% of the tap root with limited lateral roots; ns, no significant difference in disease severity between *Fusarium avenaceum* and each of the other *Fusarium* species on a t-test. *, significant difference at *p* < 0.05; **, significant difference at *p* < 0.01; and ****, significant difference at *p* < 0.0001. The dashed lines represent the overall mean disease severity across species.

Most isolates of *F. avenaceum* collected in 2021 caused more severe root rot than did isolates of *F. equiseti*, *F. solani*, *F. redolens*, *F. culmorum,* or *F. torulosum* collected in the same year (Figure 3). Similar results were observed for the isolate collections made in 2022, with *F. avenaceum* generally causing more severe root rot than *F. acuminatum*, *F. oxysporum*, *F. solani*, *F. culmorum,* or *F. redolens*.

#### 2.3.7. Correlation and Principal Component Analysis Based on Disease Severity and Growth Parameters

**Figure 4:**
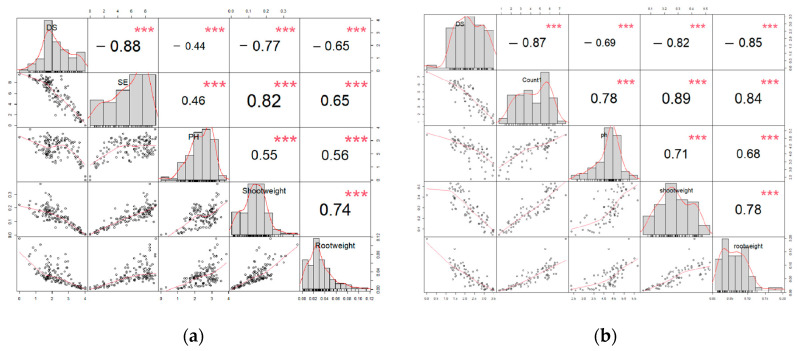
Correlation analysis between disease severity (DS), seedling emergence (SE or Count1), plant height (PH or ph), shoot dry weight (Shootweight or shootweight), and root dry weight (Rootweight or rootweight) after inoculation of the canola cv. ‘Westar’ with isolates of *Fusarium* collected in 2021 (**a**) and 2022 (**b**). Inoculations were conducted under greenhouse conditions.***, significant correlation at *p* < 0.001.

Disease severity was negatively correlated with all four plant growth parameters (emergence, plant height, shoot dry weight, root dry weight) examined in the trials conducted with both the 2021 and 2022 isolate collections (Figure 4). In contrast, emergence, plant height, shoot dry weight, and root dry weight were all positively correlated with one another.

The results of the principal component analysis (PCA) for the 2021 and 2022 growing seasons, based on all five parameters for the six most common *Fusarium* spp., are presented in Figure S4. Overall, the first two principal components (PC1 and PC2) accounted for more than 85% of the variation among all the isolates in both 2021 (86.2%) and 2022 (91.4%). In PC1, all the parameters, with the exception of plant height, showed relative contributions of approximately −0.40 for both years. Notably, plant height was the primary factor in PC2, with a relative contribution of over 0.70.

### 2.4. Phylogenetic Analysis

#### 2.4.1. Phylogenetic Tree Based on ITS Factor Sequences

A total of 143 isolates from this study (112 isolates from 2021 and 31 isolates from 2022), 13 isolates from the Applied Plant Pathology Lab collection, and one reference sequence downloaded from NCBI were included in the phylogenetic analysis. The ITS sequences obtained from these isolates ranged in size from 469 to 542 bp. Sequence alignment resulted in a sequence matrix containing 577 characters. Within this matrix, there were 365 conserved sites, 166 variable sites, and 115 parsimony-informative sites. The average composition of nucleotides was 23.0% T, 27.7% C, 25.4% A, and 23.9% G.

The 157 isolates were grouped into five distinct clades, as shown in Figure S5. Clade I comprised five isolates collected from multiple fields in 2022, including one isolate of *F. tricinctum* and four isolates of *F. avenaceum* (Figure S5). Disease severity caused by isolates in Clade I ranged from 1.89 to 3.38 (Table S2). Clade II comprised 12 isolates from multiple fields in both years, including 5 isolates of *F. acuminatum*, 6 isolates of *F. avenaceum*, and 1 isolate of *F. torulosum* (Figure S5). Disease severity caused by isolates in Clade II ranged from 1.50 to 3.50 (Table S2). Clade III consisted of 19 isolates from multiple fields in both years. This clade comprised 1 isolate of *Trichoderma paraviridescens*, 1 isolate of *F. torulosum*, and 17 isolates of *F. solani*, separated into three subgroups. The first subgroup consisted of 11 isolates of *F. solani* and 1 isolate of *F. torulosum*. The second subgroup included 6 isolates of *F. solani*, which were further divided into two sub-subgroups. The third subgroup consisted of the *T. paraviridescens* isolates (Figure S5). Disease severity caused by isolates in Clade III ranged from 0.61 to 2.54 (Table S2).

Clade IV consisted of 17 isolates from multiple fields in both years, encompassing a variety of species, including the single isolate of *Neonectria* sp., *F. oxysporum* (4 isolates), *F. equiseti* (3 isolates), *F. sporotrichioides* (2 isolates), *F. culmorum* (4 isolates), and *F. graminearum* (3 isolates). These isolates were organized into six subgroups. The first subgroup comprised two isolates of *F. graminearum* and one isolate each of *F. culmorum* and *F. oxysporum*. The second subgroup consisted of three isolates of *F. culmorum* and one isolate of *F. graminearum*. The third subgroup included two isolates of *F. sporotrichioides*. The fourth and fifth subgroups comprised three isolates each of *F. equiseti* and *F. oxysporum*, respectively. The last subgroup consisted of the *Neonectria* sp. isolate (Figure S5). Disease severity caused by isolates in Clade IV ranged from 1.48 to 4.00 (Table S2). Clade V encompassed 26 isolates from multiple fields in both years, featuring two subgroups. One subgroup contained 2 isolates of *F. proliferatum* and 1 isolate of *F. commune*, while the other subgroup included 23 *F. redolens* isolates, including the reference isolate *F. redolens* 463-5 (FRE_463) (accession number MK908825, https://www.ncbi.nlm.nih.gov/nuccore/MK908825.1) (accessed on 28 May 2023) from NCBI (Figure S5). Disease severity caused by isolates in Clade V ranged from 0.64 to 2.04 (Table S2). An additional 78 isolates from multiple fields in both years, including *F. avenaceum* (65 isolates), *F. torulosum* (9 isolates), *F. tricinctum* (1 isolate), *F. culmorum* (1 isolate), and *F. flocciferum* (2 isolates), were not grouped into any cluster. Disease severity caused by these isolates ranged from 1.22 to 3.88 (Table S2).

#### 2.4.2. Phylogenetic Tree Based on TEF-1α Sequence

The TEF-1α sequences from the 157 isolates ranged in size from 480 to 646 bp. The aligned sequence matrix contained 831 characters, consisting of 208 conserved sites, 501 variable sites, and 320 parsimony-informative sites. The average composition of nucleotides included 22.4% T, 22.4% C, 24.9% A, and 30.3% G.

The isolates were clustered into six distinct groups (Figure S6). In Clade I, 15 isolates of *F. avenaceum* from multiple fields in both years were grouped together. Two of four isolates collected near Morinville, AB, clustered as a subgroup (Figure S6). Disease severity caused by isolates in Clade I ranged from 1.60 to 3.54 (Table S2). In Clade II, 47 isolates of *F. avenaceum* from multiple fields in both years were grouped together. Among these, 32 isolates from multiple fields clustered into one subgroup, while the remaining 15 isolates from multiple fields formed another subgroup (Figure S6). Disease severity caused by isolates in Clade II ranged from 1.55 to 3.88 (Table S2). Clade III comprised seven isolates from multiple fields in both years. Within this clade, five isolates of *F. acuminatum*, one isolate of *F. avenaceum*, and one isolate of *F. tricinctum* clustered into three subgroups, respectively (Figure S6). Disease severity caused by isolates in Clade III ranged from 1.50 to 2.16 (Table S2). In Clade IV, 13 isolates from multiple fields in both years were grouped together. Within this group, two isolates of *F. flocciferum*, one isolate of *F. tricinctum*, and two isolates of *F. torulosum* were clustered into two subgroups, respectively. The remaining eight *F. torulosum* isolates clustered into three different subgroups (Figure S6). Disease severity caused by isolates in Clade IV ranged from 1.42 to 2.58 (Table S2).

In Clade V, 29 isolates from multiple fields in both years, including 22 isolates of *F. redolens*, 4 isolates of *F. oxysporum*, 2 isolates of *F. proliferatum*, and 1 isolate of *F. commune*, were grouped together. Within this group, 22 isolates of *F. redolens* from multiple fields in both years clustered in the first subgroup. The second subgroup comprised 4 isolates of *F. oxysporum*, and the third subgroup included 2 isolates of *F. proliferatum* and 1 isolate of *F. commune* (Figure S6). Disease severity caused by isolates in Clade V ranged from 0.64 to 2.45 (Table S2). Clade VI included 34 isolates from multiple fields in both years, forming nine subgroups. The first subgroup consisted of four isolates of *F. culmorum*. The second and third subgroups each included one isolate of *F. culmorum* and three isolates of *F. graminearum*, respectively. The fourth subgroup comprised two isolates of *F. sporotrichioides*, while the fifth subgroup consisted of three isolates of *F. equiseti*. The sixth subgroup comprised one isolate each of *Neonectria* sp. and *T. paraviridescens*, and the *F. redolens* isolate 463-5 (FRE_463) (accession number MK922537, https://www.ncbi.nlm.nih.gov/nuccore/MK922537.1) (accessed on 28 May 2023) from NCBI. The seventh and eighth subgroups contained 4 and 2 isolates of *F. solani*, respectively, and the ninth subgroup comprised 12 isolates of *F. solani* (Figure S6). Disease severity caused by isolates in Clade VI ranged from 0.61 to 4.00 (Table S2). An additional 12 isolates of *F. avenaceum* from multiple fields in both years were not grouped into any clade and caused disease severities ranging from 1.22 to 3.55 (Table S2).

#### 2.4.3. Phylogenetic Tree Based on the Concatenated TEF-1α and ITS Sequences

To obtain concatenated sequences, the aligned ITS and TEF-1α sequences obtained from the 157 isolates were trimmed with several forward and backward characters deleted and concatenated using MEGA 3.0. The resulting concatenated aligned sequence matrix comprised 1402 characters, with 573 conserved sites, 667 variable sites, and 435 parsimony-informative sites. The average nucleotide composition was 22.6% T, 24.8% C, 25.1% A, and 27.5% G.

**Figure 5:**
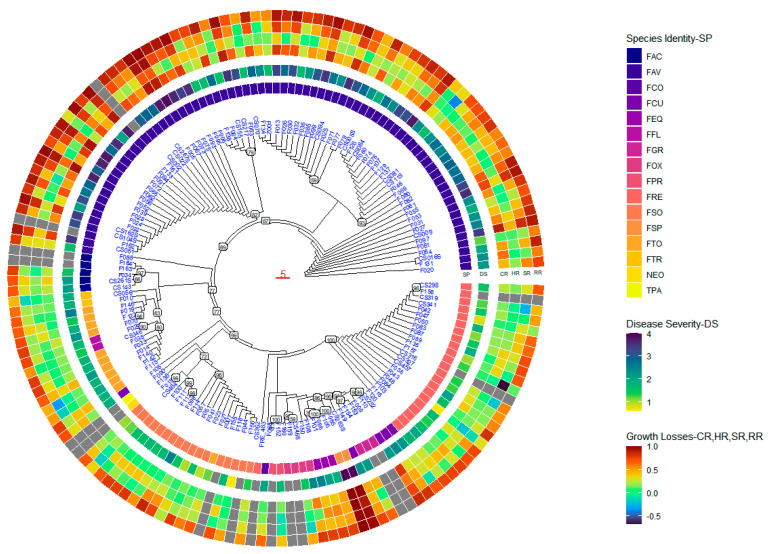
Maximum parsimony tree based on the concatenated internal transcribed spacer (ITS) and elongation factor (EF1-α) sequences from 157 fungal isolates, including 112 isolates recovered from canola in 2021 (F002-F146), 31 isolates recovered from canola in 2022 (CS002-CS466), 13 reference isolates from a laboratory culture collection (FP, F153-F164), and sequences from 1 *Fusarium redolens* isolate FRE_463 retrieved from GenBank, National Center for Biotechnology Information (NCBI). SP—species identity; FAC—*Fusarium acuminatum*; FAV—*Fusarium avenaceum*; FCO—*Fusarium commune*; FCU—*Fusarium culmorum*; FEQ—*Fusarium equiseti*; FFL—*Fusarium flocciferum*; FGR—*Fusarium graminearum*; FOX—*Fusarium oxysporum*; FPR—*Fusarium proliferatum*; FRE—*Fusarium redolens*; FSO—*Fusarium solani*; FSP—*Fusarium sporotrichioides*; FTO—*Fusarium torulosum*; FTR—*Fusarium tricinctum*; NEO—*Neonectria* sp.; and TPA—*Trichoderma paraviridescens*. DS—disease severity rated on a 0 to 4 scale [42] where 0 = healthy roots and 4 = tap root severely girdled, brown lesions on >75% of the tap root with limited lateral roots. Growth losses, reductions in seedling emergence (CR), plant height (HR), shoot dry weight (SR), and root dry weight (RR) following inoculation with each fungal isolate and relative to the corresponding non-inoculated control.

The 157 isolates were grouped into six clades in the concatenated tree (Figure 5). Clade I comprised 30 isolates of *F. avenaceum* from multiple fields of both years, in which two subgroups were included. In the first subgroup, 15 isolates were divided further into two subgroups. The second subgroup comprised another 15 isolates from multiple fields (Figure 5). Disease severity caused by isolates in Clade I ranged from 1.60 to 3.73 (Figure 4 and Table S2). Clade II consisted of 32 isolates of *F. avenaceum* from multiple fields in both years, divided into two subgroups. The first subgroup included 6 isolates from multiple fields in both years, while the second subgroup comprised 26 isolates from multiple fields in both years (Figure 5). Disease severity caused by isolates in Clade II ranged from 1.55 to 3.88 (Figure 5 and Table S2). Clade III included 20 isolates from multiple fields in both years, divided into five subgroups. The first subgroup comprised two isolates of *F. flocciferum* and six isolates of *F. torulosum*, forming two sub-subgroups, respectively. The second subgroup consisted of four isolates of *F. torulosum* and one isolate of *F. tricinctum*. The third subgroup included one isolate of *F. tricinctum*. The fourth subgroup comprised five isolates of *F. acuminatum*, with two and three isolates, respectively, clustering together. The last subgroup included one isolate of *F. avenaceum* (Figure 5). Disease severity caused by isolates in Clade III ranged from 1.42 to 2.58 (Figure 5 and Table S2).

Clade IV consisted of 21 isolates from multiple fields in both years, divided into five subgroups. The first subgroup comprised 11 isolates of *F. solani* and 1 *F. torulosum* isolate. The second subgroup included isolates of *F. solani* divided into two different clusters. One isolate each of *F. culmorum*, *Neonectria* sp., and *T. paraviridescens* were separated into three different subgroups (Figure 5). Disease severity caused by isolates in Clade IV ranged from 0.61 to 2.54 (Figure 5 and Table S2). In Clade V, 42 isolates representing eight species from multiple fields were grouped together with three subgroups. In the first subgroup, four isolates of *F. culmorum* were divided into two clusters, four isolates of *F. oxysporum* were divided into two clusters, three isolates each of *F. equiseti* and *F. graminearum* clustered into two individual clusters, and two isolates each of *F. proliferatum* and *F. sporotrichioides* were divided into individual clusters. One isolate of *F. commune* represented the last cluster. In the second subgroup, 22 isolates of *F. redolens* from multiple fields were grouped together. The *F. redolens* reference isolate 463-5 (FRE_463) clustered alone as the third subgroup (Figure 5). Disease severity caused by isolates in Clade V ranged from 0.64 to 4.00 (Figure 5 and Table S2). Another 12 isolates of *F. avenaceum* from multiple fields in both years formed an outgroup in the phylogenetic tree (Figure 4) with disease severity ranging from 1.22 to 3.55 (Figure 5 and Table S2).

### 2.5. Geographic Origins and Phylogenetic Evolution

No correlation was observed between the geographic origins and phylogenetic evolution in the phylogenetic tree, nor between the years collected and phylogenetic evolution. Most isolates that induced disease severity > 3.0 clustered in Clades I–II, consisting of *F. avenaceum* isolates (Figure 5). Additionally, two *F. sporotrichioides* isolates from Clade III caused the highest disease severity. Many isolates in Clades I–VI also caused significant reductions in seedling emergence, plant height, and shoot dry weight. However, isolates that induced high reductions in root dry weight were generally distributed across different clades (Figure 5).

## 3. Discussion

A proper understanding of the identity, diversity, and pathogenicity of common fungi can serve as the foundation for effective plant disease management strategies [43,44]. As an emerging disease, canola root rot has garnered increased attention in recent years [24,25]. Unfortunately, the last comprehensive etiological study of the fungi associated with the canola RRC in Alberta was carried out with collections made in 2009, 2010, and 2011 [14]. As such, current information on the identity and prevalence of important root rot pathogens in this province is lacking. The present research indicated that *Fusarium* spp. were the predominant fungi in the RRC during 2021–2022, with an average recovery rate of >80% from roots and >60% from stems across the surveyed fields. Other important canola pathogens recovered in this study included *R. solani* and *L. maculans*.

Over 98% (149 of 151) of the isolates recovered in 2021, and ca. 95% (75 of 79) of the isolates recovered in 2022 were confirmed as *Fusarium* spp. *Fusarium avenaceum* was most common among the species found, representing 449.7% (74 of 149) and 48% (36 of 75) of the *Fusarium* isolates in 2021 and 2022, respectively. In addition, *F. redolens* (18 isolates in 2021 and 7 isolates in 2022) and *F. solani* (14 isolates in 2021 and 8 isolates in 2022) were also fairly common compared with other species. *Fusarium torulosum* (12 isolates) was frequently isolated in 2021, but *F. oxysporum* (7 isolates) was more frequent in 2022. Three species, *F. commune*, *F. equiseti*, and *F. sporotrichioides*, were identified only in 2021. To our knowledge, this is the first report of *F. sporotrichioides* and *F. commune* causing root rot of canola anywhere in the world, although the former has been identified from *Brassica carinata* [21]. Collectively, these findings emphasize considerable species-level diversity among *Fusarium* populations extracted from diseased canola tissues, with *F. avenaceum* emerging as both the most abundant and widely distributed species. Additional species, including *F. solani*, *F. redolens*, *F. oxysporum*, and *F. torulosum*, also exhibited a wide distribution. The identification of some species only in certain years suggests that less common components of the pathogen population may sometimes go undetected. Overall, there was no correlation between field location and species.

*Fusarium roseum* (Schwein.) Petch was identified as the causal agent of canola root rot in the early 1970s [45], while *F. avenaceum* was first reported as causing canola seedling blight in the early 1980s [13]. In a more recent study, *F. avenaceum* was also identified as a highly aggressive pathogen causing seedling blight of canola, although the predominant species observed was *F. acuminatum* along with *F. culmorum*, *F. equiseti*, *F. redolens*, and *F. torulosum* [14]. In reference to other field crops, *Fusarium* spp. have been documented extensively as a cause of root rot in soybean [46,47], chickpea [48], lupine [49,50,51], wheat [52], barley [19,53,54], as well as pea and lentil [55]. These crops are commonly suggested as rotation options with canola to achieve sustainable production and mitigate root rot disease [56,57]. However, the significant species-level diversity of *Fusarium* recovered from canola, coupled with the wide host range of many of these pathogens, highlights the potential limitations of crop rotation for the management of canola root rot. For instance, the predominant pathogen identified in this study, *F. avenaceum*, was also reported to be one of the most aggressive and common species causing root rot of pea [58,59] and a cause of significant disease in chickpea, dry bean, faba bean, and lentil [42,48,58,59].

Bioassays to evaluate fungal pathogenicity offer a direct means of estimating the visual damage inflicted on the host [60], while monitoring plant growth parameters aids in understanding the pathogen’s impact on host development [61]. In this study, the pathogenicity testing included assessments of disease severity, seedling emergence, plant height, and shoot and root dry weights after inoculation of canola with isolates of *Fusarium*. The results showed a wide range of root rot severities, spanning from 0.58 to 4.0. However, the majority of isolates (159 out of 205 caused moderate to severe root rot, with disease severities ranging from 1.0 to 3.0. Another 40 isolates, consisting of *F. avenaceum* and *F. sporotrichioides*, caused very severe disease (>3.0). Earlier studies have reported that *F. avenaceum*, *F. solani*, and *F. equiseti* are causing the most severe symptoms on canola [14,24].

Similarly, the majority of isolates had notable effects on the growth of the canola plants. Isolates of *F. avenaceum*, along with a few isolates of *F. solani*, *F. redolens*, *F. culmorum*, *F. equiseti*, and *F. sporotrichioides*, stood out as among the most aggressive in reducing root and shoot biomass. Seedling emergence was also reduced by more than 70% of the isolates, with *F. avenaceum*, *F. solani*, *F. sporotrichioides*, and *F. culmorum* identified as the most aggressive species. In contrast, plant height was less affected, with a third of the isolates not having a significant effect on this parameter. Indeed, one isolate each of *F. solani* and *F. tricinctum* significantly increased plant height, and they did not reduce shoot dry weight or seedling emergence. This might indicate that these isolates have some beneficial functions in promoting plant growth. Both *F. solani* and *F. tricinctum* have been reported as potentially beneficial endophytic fungi in plants in responding to biotic stress [62,63].

Correlation analysis indicated a robust negative association between disease severity and all plant growth parameters evaluated. In a previous study, a similar negative correlation was observed between disease severity and canola seedling emergence after co-inoculation with *F. proliferatum* and *F. oxysporum* [8]. In contrast, all four growth parameters exhibited positive correlations with each other. Madden et al. recommended principal component analysis (PCA) for quantitatively assessing plant disease epidemics [64]. In this study, PC1 and PC2 accounted for over 85% of the overall variation among all isolates. Additionally, disease severity and the four growth parameters all emerged as major contributors to PC1, while root dry weight and plant height were important in PC2. The use of correlation analysis and PCA of disease severity and growth parameters allows for a more comprehensive evaluation of pathogenicity, disease development, and host growth characteristics.

With the advancement of molecular biology techniques, sequencing-based analyses have emerged as valuable tools in taxonomic studies, supplementing traditional phenotypic and morphological methods [65,66]. In the present study, three distinct phylogenetic trees were constructed based on the fungal ITS sequence, TEF-1α sequence, and the concatenated ITS and TEF-1α sequences. The broad topological structures of these phylogenetic trees exhibited similarity and effectively depicted the phylogenetic relationships within the *Fusarium* genus. Notably, the phylogenetic tree generated from the ITS sequences highlighted distinct clustering for most species, with the exception of genetically related species such as *F. avenaceum*, *F. tricinctum*, *F. torulosum*, and *F. acuminatum*, which were grouped together. This was anticipated, as these four species constitute part of the *Fusarium tricinctum* species complex (FTSC), which has emerged as a disease issue in small grain cereals and other crops [67,68]. Similar findings were reported in previous phylogenetic investigations of *Fusarium* species associated with canola seedling blight [14], as well as across species originating from diverse geographical locations [65].

The TEF-1α sequence analysis resulted in six distinct clades, effectively distinguishing all the *Fusarium* species. Additionally, the construction of the phylogenetic tree based on the concatenated sequences of the ITS and TEF-1α regions effectively organized the *Fusarium* species into distinct clades. Across all three phylogenetic trees, the data consistently indicated a relatively low diversity within *F. avenaceum*, aligning with findings from previous studies [14,59]. While a close phylogenetic relationship was observed among *F. avenaceum*, *F. tricinctum*, *F. torulosum*, and *F. acuminatum*, highlighting their genetic similarity, *F. redolens,* and *F. oxysporum* exhibited relatively greater genetic distance from *F. avenaceum*. No correlations were found between the phylogenetic grouping and geographic origin, year of isolation, or pathogenicity characteristics of the isolates. This may reflect the relatively restricted geographic scale of the survey, which was limited to northcentral Alberta, and the fact that samples were collected over two consecutive years. The phylogenetic tree based on concatenated sequences not only facilitated clear differentiation between the various species but also allowed further classification within species, as exemplified by *F. avenaceum*. This suggests that employing concatenated sequences in phylogenetic tree construction is a superior strategy, particularly for species with limited genetic diversity. Similarly, concatenated analyses of multi-locus DNA sequence data are widely recognized as a powerful and commonly used method for exploring evolutionary and phylogenetic relationships between isolates, for example, with the *Fusarium oxysporum* species complex [69]. In an earlier report, the enhanced discriminatory capacity of the combined phylogenetic tree was particularly pronounced for species like *F. avenaceum*, *Fusarium arthrosporioides* Sherb., and *F. tricinctum*, which exhibit morphological similarities [65].

In conclusion, this study addressed the emerging challenge of canola root rot via a comprehensive investigation of its causal agents and their impact on plant growth. *Fusarium* spp. represent an important component of the canola RRC, with this study suggesting that *F. avenaceum* is the predominant and most aggressive species. Principal component analysis and correlation analysis confirmed the necessity of conducting a comprehensive assessment of pathogenicity, taking into account both disease severity and growth parameters. Phylogenetic analysis, based on the TEF-1α sequences and the concatenated ITS and TEF-1α sequences, offered insights into the genetic relationships among *Fusarium* species, with both sequences proving particularly effective in discerning closely related populations. The high species-level diversity of *Fusarium* spp. involved in canola root rot also suggests that single methods, such as crop rotation, may not be sufficient for disease control. This underscores the need for integrated strategies for root rot management. The findings provide an update on the status of the canola RRC in Alberta and pave the way for more targeted control measures to mitigate its impact on crop production.

## 4. Materials and Methods

### 4.1. Disease Surveys

#### 4.1.1. Root Rot Surveys

Canola crops in north-central Alberta were surveyed for the occurrence of root rot in late August to October 2021 [40] and September 2022 [41], following a W-shaped transect in each field. A total of 35 and 51 fields were visited in each of 2021 and 2022, respectively. Symptomatic root and lower stem samples, including those displaying brown girdling root rot, foot, and root rot, or brown, soft, sunken lesions [70], were collected for processing in the laboratory. The incidence of root rot was calculated as the percentage of diseased samples relative to the total collected in each field, with five 1-m^2^ quadrats sampled per field along a W-transect [40,41]. The surveyed region is illustrated in Figure 1 and Figure S1, as well as Table S1.

#### 4.1.2. Fungal Isolate Recovery

A total of 3842 symptomatic root and stem samples were cultured for pathogen recovery. This number included 524 root and 402 stem samples collected in 2021 and 1871 root and 1045 stem samples collected in 2022. In brief, samples were surface sterilized in 1% bleach (NaOCl) for 2 min and rinsed 3× in sterile distilled water. The rinsed samples were incubated on potato dextrose agar (PDA) at room temperature under a 12-h cycle of ambient light for 10–12 days. Subsequently, the recovered pathogens were transferred to a 2% water agar medium and purified using the hyphal tip method [71]. Recovered pathogens were identified at the genus and/or species level by examining the colony and morphological characteristics of each colony, such as the sclerotia produced by *R. solani* [72] and the colony color and microscopic structures (macroconidia, microconidia, chlamydospores, and phialides) produced by species of *Fusarium* [30].

### 4.2. Isolation and Molecular Identification of Fusarium Species

#### Hyphal Tip Purification and Species Identification

The *Fusarium* isolates were tentatively identified based on the colony, and morphological characteristics as described above were sub-cultured for hyphal tip purification [73], and their identities were confirmed by molecular analysis. Purified isolates were cultured on PDA for 7–10 days, as above, and stored at 4 °C for future use. The DNA of 230 selected isolates, including 151 isolates recovered from samples collected in 2021 and 79 isolates recovered from samples collected in 2022, was extracted from 7-day-old PDA cultures following the Quick-Start protocol (www.qiagen.com/HB-2552) (accessed on 25 February 2022) of the DNeasy Plant Pro Kit (Qiagen, Hilden, Germany). DNA concentration and quality were assessed with a NanoDrop 2000 spectrophotometer (Thermo Fisher Scientific, Toronto, ON, Canada) using the default setting for a DNA assay. The samples were then diluted to a final concentration of 20 ng DNA/μL with nuclease-free water (Thermo Fisher Scientific) and stored at −20 °C until needed.

The DNA extracted from each of the 230 fungal isolates was subjected to PCR using two sets of primers: EF-2 (5′-GGARGTACCAGTSATCATGTT-3′) and EF-3 (5′-GTAAGGAGGASAAGACTCACC-3′), targeting the TEF-1α gene [74]; and ITS4 (5′-TCCTCCGCTTATTGATATGC-3′) and ITS5 (5′-GGAAGTAAAAGTCGTAACAAGG-3′) [75], targeting the ITS region. Reactions were conducted in a total volume of 20 μL, which included 1 μL DNA template (20 ng/μL), 1 μL of each forward and reverse primer (10 μM), 10 μL HotStar Taq Master Mix (Qiagen), 0.25 μL bovine serum albumin (10 mg/mL) (Thermo Fisher Scientific), and 6.75 μL nuclease-free water (Thermo Fisher Scientific). Thermocycler conditions consisted of an initial heat activation at 95 °C for 5 min, followed by 35 cycles of denaturation at 94 °C for 1 min, annealing at 52 °C for 30 sec (marked with 55 °C for 90 sec for the EF-2/EF-3 primer pair), and extension at 72 °C for 1 min. A final extension step was conducted at 72 °C for 10 min. Amplicons were purified with a QIAquick PCR purification kit (Qiagen) according to the manufacturer’s instructions and sequenced via Sanger sequencing [76] at the Molecular Biology Service Unit (MBSU) of the University of Alberta, Edmonton, Canada.

The TEF-1α and ITS sequences obtained were used to confirm the species classification of each isolate via BLAST searches (https://blast.ncbi.nlm.nih.gov/Blast.cgi) (accessed on 25 May 2023) of the National Center for Biotechnology Information (NCBI) nucleotide databases. Morphological characterization and molecular identification were compared to determine the final identity of the isolates at the species level.

### 4.3. Pathogenicity Test

The pathogenicity of 205 *Fusarium* isolates (130 isolates collected in 2021 and 75 isolates collected in 2022) was assessed on the canola cv. ‘Westar’ under greenhouse conditions. Briefly, 473 mL cups (Uline, Toronto, ON, Canada) were filled with 400 mL of Promix PGX potting medium (Sun-Gro Canada Inc., Seba Beach, AB, Canada). Fourteen-day-old fungal cultures produced on PDA were sectioned into small pieces (3 mm × 3 mm) using a sterilized scalpel and then spread evenly on the surface of the potting mix within each cup (at a rate of one colony or plate per cup) [77]. Ten ‘Westar’ seeds were planted in each cup, with four cups (replicates) allocated for each isolate (treatment). An equal number of cups was designated for the non-inoculated control, with the same quantity of PDA pieces distributed alongside the seeds. Two trials were conducted, with treatments arranged in a randomized complete block design. The plants were watered and fertilized (20N:20P:20K) as needed.

Following inoculation, the cups were transferred to a greenhouse maintained at 25 °C with a 12-h photoperiod. Seedling emergence data were collected on the 7th day after seeding, while plant height was assessed on the 14th day. On the 21st day after seeding, the plants were uprooted, and their roots were thoroughly washed with tap water before being assessed for disease severity, as described below. Subsequently, the plants were air-dried at 25 °C for 7 days, and the shoots and roots were separated by cutting. Shoot and root dry weights were determined for each experimental unit.

### 4.4. Disease Ratings

Root rot severity was assessed on a 0–4 scale [42], where 0 = healthy roots; 1 = small, light-brown lesions on <25% of the tap root; 2 = brown lesions on 25–49% of the tap root; 3 = brown lesions on 50–74% of the tap root, tap root constricted; and 4 = tap root severely girdled, brown lesions on >75% of the tap root with limited lateral roots. The final disease severity per experimental unit was determined by averaging the values of all the individual plants within each cup (replicate).

### 4.5. Emergence, Plant Height, and Shoot and Root Dry Weights

Seedling emergence was determined by counting all surviving plants in each experimental unit 7 days after seeding. Plant height was measured from the soil line to the shoot apex using a ruler 14 days after seeding. Shoot and root dry weights were assessed separately for each replicate with a weighing scale (Fisher Science Education SLF 303, Thermo Fisher). Reductions in seedling emergence, plant height, and shoot and root dry weights were calculated relative to non-inoculated controls according to the following equation:

Reduction = [(D_ck_ − D_tr_)/D_ck_] × 100%, (1)

where D_ck_ represents the control (non-inoculated) treatment and D_tr_ represents the inoculated treatment.

### 4.6. Phylogenetic Tree Construction

Three independent phylogenetic trees were constructed for 157 isolates based on their (i) ITS sequence, (ii) TEF-1α sequence, and (iii) the concatenated ITS and TEF-1α sequences. These included 112 representative isolates collected in 2021 (110 *Fusarium* isolates and 1 isolate each of *T. paraviridescens* and *Neonectria* sp.), 31 representative *Fusarium* isolates collected in 2022, one reference sequence from NCBI, and 13 isolates from the culture collection of the Applied Plant Pathology Lab, University of Alberta. The latter 13 cultures consisted of one isolate of *F. proliferatum* and two isolates each of *F. graminearum*, *F. solani*, *F. redolens*, *F. oxysporum*, *F. avenaceum*, and *F. acuminatum*. Sequence characters were equally weighted in all the phylogenetic analyses, with alignment performed using the MUSCLE algorithm in MEGA 3.0 [78]. Maximum parsimony (MP) trees were generated with default parameters and bootstrapping using 1000 replicates. All phylogenetic trees were reconstructed using R Studio v. 4.1.2 with the ggtree package [79]. Species identity was assigned to isolates in the ITS and TEF-1α sequence trees. In the concatenated sequence tree, each isolate was assigned species identity, along with disease severity and reductions in emergence, root dry weight, and shoot dry weight. Consistency among the single-locus phylogenetic trees and the combined gene phylogeny was evaluated based on the overall topology of each tree [80].

### 4.7. Data Analysis

All datasets were evaluated for homogeneity of the variance by ANOVA using R Studio v. 4.1.2 [79]. Differences were considered significant when the *p*-value was <0.05 unless otherwise indicated. In cases where a significant interaction was detected between repetition and treatment, the data were analyzed separately to account for the effects of these factors on the results. In the pathogenicity test, a *t*-test was employed using R Studio v. 4.1.2 [79] to compare the non-inoculated control and inoculated treatments. Correlation analysis was conducted to assess the relationship between disease severity and plant growth parameters. Principal component analysis (PCA) was used to explore the potential utility of parameters for evaluating pathogenicity. Root rot severity and reductions in seedling emergence, plant height, and root and shoot dry weight were used to generate the PCA biplot. Phylogenetic trees were constructed in MEGA 3.0 and then modified in R Studio with the ggtree package.

## Figures and Tables

**Table 1:** Root rot severity and reductions in emergence and plant height following inoculation of the canola cv. ‘Westar’ with isolates representing different *Fusarium* species under greenhouse conditions.

Species ^a^	Disease Severity (0–4) ^b^					Reduction in Emergence ^c^ (%)					Reduction in Height ^d^ (%)				
	Range	0–1.0	1.01–2.0	2.01–3.0	3.01–4.0	Range	<25	25.1–50	50.1–75	>75	Range	<25	25.1–50	50.1–75	>75
FAC	1.50–2.41	0	3	3	0	19.7–37.7	4	2	0	0	−1.9–17.7	6	0	0	0
FAV	1.22–3.88	0	17	51	37	10.5–93.4	11	31	39	24	−8.6–71.6	66	28	11	0
FCO	2.04	0	0	1	0	10.5	1	0	0	0	14.70	1	0	0	0
FCU	1.46–2.37	0	5	5	0	13.1–80.3	4	5	0	1	10.1–60.0	3	6	1	0
FEQ	1.86–2.52	0	1	3	0	38.2–50.0	0	4	0	0	47.9–57.5	0	1	3	0
FFL	1.42–1.72	0	2	0	0	16.4–17.1	2	0	0	0	6.9–8.9	2	0	0	0
FGR	2.19	0	0	1	0	44.70	0	1	0	0	2.40	1	0	0	0
FOX	1.48–2.45	0	4	4	0	14.8–59.2	1	4	3	0	2.5–33.1	7	1	0	0
FPR	1.71	0	1	0	0	14.5	1	0	0	0	−9.7	1	0	0	0
FRE	0.58–2.04	3	21	1	0	1.3–52.5	19	5	1	0	−18.6–47.2	14	11	0	0
FSO	0.61–2.54	3	16	2	0	2.6–77.0	18	2	0	1	−5.3–54.9	13	7	1	0
FSP	4	0	0	0	2	100	0	0	0	2	91.2–100	0	0	0	2
FTO	1.19–2.58	0	10	3	0	6.6–44.7	10	3	0	0	−4.3–40.7	8	5	0	0
FTR	1.78–1.96	0	3	0	0	11.5–23.0	3	0	0	0	−8.6–17.1	3	0	0	0
Fsp	2.41–3.48	0	0	2	1	29.5–73.8	0	1	2	0	17.6–35.0	2	1	0	0
Total	0.58–4.0	6	83	76	40	1.3–100	74	58	45	28	−18.6–100	127	60	16	2

^a^ FAC—Fusarium acuminatum; FAV—Fusarium avenaceum; FCO—Fusarium commune; FCU—Fusarium culmorum; FEQ—Fusarium equiseti; FFL—Fusarium flocciferum; FGR—Fusarium graminearum; FOX—Fusarium oxysporum; FPR—Fusarium proliferatum; FRE—Fusarium redolens; FSO—Fusarium solani; FSP—Fusarium sporotrichioides; FTO—Fusarium torulosum; FTR—Fusarium tricinctum; and Fsp—unidentified species. ^b^ Root rot disease severity (0–4 scale) [42] at 21 days after seeding; the number of isolates of each species under each set of values is indicated. Control (non-inoculated) treatments did not develop any disease (severity = 0). ^c^ Reduction in seedling emergence in the inoculated treatment relative to the non-inoculated control; the number of isolates of each species under each set of values is indicated. ^d^ Reduction in plant height at 14 days after seeding in the inoculated treatment relative to the non-inoculated control; the number of isolates of each species under each set of values is indicated.

**Table 2:** Reductions in shoot and root dry weights of canola cv. ‘Westar’ following inoculation with isolates representing different *Fusarium* species under greenhouse conditions.

Species ^a^	Shoot Dry Weight Reduction ^b^ (%)					Root Dry Weight Reduction ^c^ (%)				
	Range	<25	25.1–50	50.1–75	>75	Range	<25	25.1–50	50.1–75	>75
FAC	−26.5–37.3	3	3	0	0	55.8–77.7	0	0	5	1
FAV	−38.9–94.9	15	37	28	25	30.6–98.2	0	6	24	75
FCO	30.5	0	1	0	0	63.5	0	0	1	0
FCU	−19.6–75.2	5	3	1	1	18.8–84.9	1	0	5	4
FEQ	42.5–54.2	0	2	2	0	78.0–84.1	0	0	0	4
FFL	5.3–24.9	2	0	0	0	54.5–75.1	0	0	1	1
FGR	24.7	1	0	0	0	64.7	0	0	1	0
FOX	−13.7–47.2	4	4	0	0	45.5–79.7	0	2	5	1
FPR	−6.7	1	0	0	0	41.2	0	1	0	0
FRE	−67.9–52.2	15	9	1	0	11.9–82.2	2	3	15	5
FSO	−24.3–58.8	15	3	3	0	23.3–87.7	1	2	11	7
FSP	96.4–98.6	0	0	0	2	96.6–99.6	0	0	0	2
FTO	−33.7–43.9	10	3	0	0	46.9–79.1	0	2	6	5
FTR	−12.8–20.3	3	0	0	0	43.9–62.6	0	1	2	0
Fsp	27.3–82.5	0	2	0	1	61.2–93.9	0	0	2	1
Total	−67.9–98.6	74	67	35	29	11.9–99.6	4	17	78	106

^a^ FAC—Fusarium acuminatum; FAV—Fusarium avenaceum; FCO—Fusarium commune; FCU—Fusarium culmorum; FEQ—Fusarium equiseti; FFL—Fusarium flocciferum; FGR—Fusarium graminearum; FOX—Fusarium oxysporum; FPR—Fusarium proliferatum; FRE—Fusarium redolens; FSO—Fusarium solani; FSP—Fusarium sporotrichioides; FTO—Fusarium torulosum; FTR—Fusarium tricinctum; and Fsp—unidentified species. ^b^ Reduction in shoot dry weight in the inoculated treatment relative to the non-inoculated control at 21 days after seeding; the number of isolates of each species under each set of values is indicated. ^c^ Reduction in root dry weight in the inoculated treatment relative to the non-inoculated control at 21 days after seeding; the number of isolates of each species under each set of values is indicated.

## Data Availability

The data presented in this study are available from the corresponding authors upon request.

## Supplementary Materials

The supporting information can be downloaded at https://www.mdpi.com/article/10.3390/ijms25116244/s1.
