# Supplementary material for: Diversity and Pathogenicity of Fusarium Root Rot Fungi from Canola (Brassica napus) in Alberta, Canada
Source: Int J Mol Sci. 2024 Jun 5;25(11):6244. doi: 10.3390/ijms25116244 (PMC11172839; doi:10.3390/ijms25116244)
Supplement: Supplementary file 1 [file ijms-25-06244-s001.zip › ijms-3004946-supplementary.pdf]

Table S1. Location of fields surveyed for the incidence of canola root rot in 2021 and 2022.

Field No.[a]	Location	Field No.[a]	Location	Field No.[a]	Location	Field No.[a]	Location
2021-1	St. Albert	2021-24	Morinville	2022-12	Leduc	2022-35	Lamont
2021-2	St. Albert	2021-25	Morinville	2022-13	Leduc	2022-36	Thorhild
2021-3	St. Albert	2021-26	Redwater	2022-14	Leduc	2022-37	Thorhild
2021-4	St. Albert	2021-27	Opal	2022-15	Leduc	2022-38	Thorhild
2021-5	Edmonton	2021-28	Bruderheim	2022-16	Parkland	2022-39	Thorhild
2021-6	Edmonton	2021-29	Bruderheim	2022-17	Parkland	2022-40	Thorhild
2021-7	Edmonton	2021-30	Bruderheim	2022-18	Parkland	2022-41	Beaver
2021-8	Edmonton	2021-31	Edmonton	2022-19	Parkland	2022-42	Beaver
2021-9	Edmonton	2021-32	Edmonton	2022-20	Parkland	2022-43	Beaver
2021-10	Morinville	2021-33	Redwater	2022-21	Westlock	2022-44	Beaver
2021-11	Morinville	2021-34	Redwater	2022-22	Westlock	2022-45	Beaver
2021-12	Morinville	2021-35	Redwater	2022-23	Westlock	2022-46	Flagstaff
2021-13	Morinville	2022-1	Sturgeon	2022-24	Westlock	2022-47	Flagstaff
2021-14	Namao	2022-2	Sturgeon	2022-25	Westlock	2022-48	Flagstaff
2021-15	Edmonton	2022-3	Sturgeon	2022-26	Barrhead	2022-49	Flagstaff
2021-16	Gibbons	2022-4	Sturgeon	2022-27	Barrhead	2022-50	Flagstaff
2021-17	Gibbons	2022-5	Sturgeon	2022-28	Barrhead	2022-51	Flagstaff
2021-18	Josephburg	2022-6	Strathcona	2022-29	Barrhead		
2021-19	Josephburg	2022-7	Strathcona	2022-30	Barrhead		
2021-20	St. Albert	2022-8	Strathcona	2022-31	Lamont		
2021-21	Villeneuve	2022-9	Strathcona	2022-32	Lamont		
2021-22	Villeneuve	2022-10	Strathcona	2022-33	Lamont		
2021-23	Villeneuve	2022-11	Leduc	2022-34	Lamont		

[a]Isolated year and field number

Table S2. Pathogenicity of *Fusarium* isolates causing root rot and their impact on growth of canola seedlings.

Isolate	Species[a]	Disease Severity[b]	Countrd[c]	Phrd[d]	Shootrd[e]	Rootrd[f]	Field No.[g]	Tree[h]	Accession #[i]
CS139	FAC	2.41	27.9%	16.7%	27.8%	72.6%	2022-21		
CS143	FAC	2.16	24.6%	14.3%	30.5%	68.4%	2022-25	1	PP499066
CS178	FAC	1.64	19.7%	5.7%	-26.5%	57.8%	2022-18		
CS261S	FAC	2.10	37.7%	17.7%	37.3%	70.3%	2022-26	1	PP499071
F034	FAC	1.50	23.7%	-1.9%	17.0%	77.7%	2021-16	1	PP499104
F125	FAC	1.79	25.0%	11.3%	19.0%	55.8%	2021-32		
CS002	FAV	2.99	73.8%	27.3%	73.2%	87.8%	2022-11	1	PP499053
CS005S	FAV	2.24	67.2%	17.7%	61.4%	87.9%	2022-23	1	PP499054
CS009	FAV	2.22	41.0%	13.4%	41.7%	80.8%	2022-26	1	PP499055
CS016S	FAV	2.00	50.8%	13.0%	45.5%	88.7%	2022-20	1	PP499056
CS018S	FAV	2.39	49.2%	12.5%	35.9%	78.2%	2022-18		
CS020	FAV	3.13	62.3%	17.9%	55.1%	84.8%	2022-21		
CS022	FAV	2.91	63.9%	18.4%	60.2%	85.6%	2022-11	1	PP499057
CS034	FAV	2.76	67.2%	23.4%	64.6%	84.3%	2022-48		
CS048	FAV	2.90	65.6%	17.9%	44.5%	81.7%	2022-31		
CS051	FAV	3.46	77.0%	38.8%	79.4%	92.1%	2022-23	1	PP499058
CS057	FAV	1.95	37.7%	10.7%	26.4%	72.9%	2022-21		
CS070	FAV	2.88	70.5%	14.9%	71.5%	88.6%	2022-21	1	PP499060
CS081	FAV	2.28	42.6%	6.1%	30.8%	72.6%	2022-23	1	PP499061
CS084	FAV	2.69	50.8%	17.3%	39.6%	77.8%	2022-26	1	PP499062
CS085S	FAV	1.97	32.8%	14.4%	10.2%	56.5%	2022-2		
CS089	FAV	2.71	70.5%	28.6%	64.4%	86.8%	2022-15		
CS092S	FAV	3.00	83.6%	33.3%	84.3%	94.7%	2022-43		
CS123	Fsp	3.48	73.8%	35.0%	82.5%	93.9%	2022-15		

CS094	FAV	2.65	47.5%	19.4%	25.1%	81.4%	2022-37	1	PP499063
CS138S	Fsp	2.41	29.5%	17.6%	37.1%	61.2%	2022-22		
CS098S	FAV	3.46	85.2%	37.1%	80.6%	93.9%	2022-17		
CS103	FAV	3.04	80.3%	54.5%	82.0%	94.6%	2022-23		
CS104S	FAV	3.14	55.7%	17.0%	46.6%	84.8%	2022-19	1	PP499064
CS119	FAV	2.73	49.2%	10.7%	47.5%	72.7%	2022-20	1	PP499065
CS128S	FAV	3.44	90.2%	50.5%	89.6%	96.3%	2022-6		
CS145S	FAV	2.45	60.7%	15.0%	29.7%	86.0%	2022-26		
CS150	FAV	3.00	60.7%	22.1%	39.4%	89.2%	2022-26		
CS155	FAV	3.50	88.5%	43.7%	78.4%	95.8%	2022-47	1	PP499067
CS162S	FAV	3.38	80.3%	28.0%	84.6%	95.4%	2022-36	1	PP499068
CS163	FAV	3.14	73.8%	31.2%	66.6%	92.4%	2022-51		
CS185S	FAV	3.07	78.7%	46.0%	61.6%	93.2%	2022-7		
CS202	FAV	2.86	77.0%	21.6%	63.8%	92.7%	2022-38		
CS214	FAV	3.20	68.9%	23.7%	49.8%	88.1%	2022-7		
CS217	FAV	3.17	65.6%	24.9%	56.1%	70.0%	2022-48	1	PP499069
CS267S	FAV	2.85	52.5%	32.0%	54.1%	83.1%	2022-22	1	PP499073
CS219	Fsp	2.64	57.4%	23.6%	27.3%	58.7%	2022-46		
CS269S	FAV	2.88	59.0%	40.1%	45.5%	82.5%	2022-19		
CS284	FAV	2.27	23.0%	14.2%	22.4%	72.4%	2022-23		
CS355	FAV	2.06	39.3%	13.6%	44.8%	65.9%	2022-5		
F002	FAV	1.60	23.7%	14.0%	37.5%	56.0%	2021-2	1	PP499099
F005	FAV	3.75	92.1%	69.1%	92.9%	98.2%	2021-2	1	PP499124
F006	FAV	1.68	26.3%	41.3%	50.2%	75.9%	2021-3	1	PP499135
F013	FAV	3.31	80.3%	37.8%	79.5%	92.2%	2021-10	1	PP499085
F016	FAV	3.66	86.8%	63.7%	90.7%	97.1%	2021-12		
F017	FAV	3.77	89.5%	62.2%	93.6%	97.7%	2021-12		

F020	FAV	2.23	43.4%	16.9%	56.7%	76.1%	2021-13	1	PP499089
F022	FAV	2.11	32.9%	6.4%	27.9%	81.5%	2021-14	1	PP499091
F024	FAV	3.19	71.1%	10.1%	70.0%	92.2%	2021-16	1	PP499093
F025	FAV	2.30	40.8%	15.4%	36.7%	84.3%	2021-16	1	PP499094
F026	FAV	2.79	67.1%	1.7%	60.4%	86.4%	2021-16	1	PP499095
F027	FAV	1.89	26.3%	-0.5%	23.0%	71.6%	2021-16	1	PP499096
F028	FAV	2.65	50.0%	-8.6%	41.6%	83.6%	2021-16	1	PP499097
F030	FAV	3.19	71.1%	28.1%	75.1%	93.2%	2021-16	1	PP499100
F031	FAV	2.33	55.3%	-1.5%	47.3%	81.9%	2021-16	1	PP499101
F032	FAV	1.66	26.3%	1.5%	25.5%	79.1%	2021-16	1	PP499102
F036	FAV	2.24	52.6%	2.4%	47.1%	82.8%	2021-16	1	PP499106
F039	FAV	2.88	63.2%	18.0%	75.5%	90.9%	2021-19	1	PP499108
F046	FAV	2.44	52.6%	12.6%	58.2%	76.2%	2021-21	1	PP499113
F049	FAV	1.82	34.2%	19.6%	51.6%	72.0%	2021-23		
F052	FAV	3.69	93.4%	63.0%	92.1%	97.6%	2021-23	1	PP499118
F053	FAV	3.55	75.0%	33.7%	82.6%	93.0%	2021-23	1	PP499119
F054	FAV	1.22	10.5%	10.2%	17.3%	58.7%	2021-23	1	PP499120
F055	FAV	3.29	67.1%	12.1%	65.6%	91.2%	2021-24	1	PP499121
F056	FAV	2.96	47.4%	4.4%	49.5%	73.8%	2021-24	1	PP499122
F060	FAV	2.34	32.9%	28.3%	35.7%	81.8%	2021-24	1	PP499125
F061	FAV	2.87	46.1%	2.8%	55.3%	57.6%	2021-24	1	PP499126
F062	FAV	3.67	78.9%	12.3%	75.7%	87.3%	2021-24	1	PP499127
F063	FAV	1.65	21.1%	5.2%	7.7%	50.8%	2021-24	1	PP499128
F064	FAV	1.55	15.8%	9.4%	13.7%	57.8%	2021-24	1	PP499129
F066	FAV	3.75	93.4%	58.6%	90.6%	92.9%	2021-25	1	PP499131
F067	FAV	3.88	89.5%	67.4%	94.9%	97.9%	2021-25	1	PP499132
F069	FAV	2.76	40.8%	9.0%	44.6%	82.6%	2021-25	1	PP499134

F071	FAV	3.54	84.2%	31.4%	77.9%	93.9%	2021-25	1	PP499137
F072	FAV	2.81	23.7%	5.6%	13.1%	30.6%	2021-25	1	PP499138
F073	FAV	3.63	75.0%	23.4%	74.4%	93.1%	2021-25	1	PP499139
F074	FAV	3.30	57.9%	22.0%	63.0%	87.3%	2021-25	1	PP499140
F075	FAV	3.35	63.2%	10.3%	67.4%	92.2%	2021-25	1	PP499141
F076	FAV	3.15	53.9%	13.9%	59.5%	80.2%	2021-25	1	PP499142
F077	FAV	3.07	60.5%	8.2%	63.9%	70.8%	2021-25	1	PP499143
F078	FAV	2.96	22.4%	31.4%	41.9%	44.7%	2021-25		
F079	FAV	2.79	26.3%	24.8%	39.7%	45.3%	2021-25	1	PP499144
F080	FAV	2.92	42.1%	19.3%	44.1%	57.4%	2021-25	1	PP499146
F081	FAV	3.42	89.5%	45.6%	84.7%	91.5%	2021-25	1	PP499147
F082	FAV	2.75	39.5%	6.1%	41.6%	64.6%	2021-25	1	PP499148
F085	FAV	3.05	44.7%	36.4%	67.3%	89.1%	2021-26	1	PP499151
F088	FAV	1.62	31.6%	32.5%	34.6%	79.8%	2021-26	1	PP499154
F091	FAV	3.56	73.7%	57.3%	79.6%	96.5%	2021-27	1	PP499157
F093	FAV	3.54	78.9%	45.0%	80.2%	96.4%	2021-27	1	PP499158
F094	FAV	2.04	46.1%	35.5%	20.5%	78.5%	2021-27	1	PP499159
F095	FAV	1.63	18.4%	40.1%	15.1%	76.1%	2021-28	1	PP499160
F096	FAV	2.94	60.5%	47.4%	47.0%	86.8%	2021-28		
F097	FAV	2.43	57.9%	36.7%	54.9%	87.0%	2021-28	1	PP499161
F099	FAV	3.44	84.2%	49.6%	79.2%	95.3%	2021-29	1	PP499163
F106	FCU	1.92	15.8%	44.3%	32.4%	72.3%	2021-30	1	PP499170
F107	FAV	3.73	78.9%	55.3%	82.6%	91.5%	2021-30	1	PP499171
F124	FAV	1.88	30.3%	20.2%	32.6%	68.8%	2021-32		
F126	FAV	1.85	18.4%	23.4%	31.5%	59.3%	2021-32	1	PP499182
F127	FAV	2.34	47.4%	22.9%	40.3%	71.5%	2021-32		
F128	FAV	2.13	53.9%	33.7%	43.4%	76.4%	2021-32		

F129	FAV	2.35	32.9%	9.5%	5.1%	38.8%	2021-32	1	PP499183
F131	FAV	2.33	61.8%	5.4%	12.5%	59.1%	2021-32	1	PP499184
F133	FAV	3.73	90.8%	71.6%	85.5%	96.5%	2021-32	1	PP499185
F134	FAV	2.33	43.4%	12.2%	1.1%	51.2%	2021-33	1	PP499186
F137	FAV	1.61	18.4%	-0.9%	-38.9%	34.3%	2021-33	1	PP499189
F138	FAV	2.79	78.9%	24.4%	53.5%	89.5%	2021-33	1	PP499190
F141	FAV	1.89	15.8%	-0.8%	-27.3%	34.7%	2021-33		
F143	FAV	2.10	30.3%	6.8%	13.7%	62.1%	2021-33		
F144	FAV	2.59	68.4%	2.4%	44.0%	81.5%	2021-33		
F145	FAV	2.56	52.6%	5.7%	38.6%	84.7%	2021-33		
F068	FCO	2.04	10.5%	14.7%	30.5%	63.5%	2021-25	1	PP499133
CS246S	FCU	1.76	13.1%	10.1%	-19.6%	65.0%	2022-34		
CS248S	FCU	1.78	24.6%	21.7%	-11.4%	66.6%	2022-32		
CS250	FCU	1.46	27.9%	18.9%	9.3%	18.8%	2022-37		
CS252	FCU	2.22	26.2%	29.1%	-9.4%	67.7%	2022-12	1	PP499070
CS263S	FCU	2.27	47.5%	41.9%	50.9%	75.1%	2022-48	1	PP499072
F009	FCU	2.19	80.3%	60.0%	75.2%	84.9%	2021-3	1	PP499164
F109	FCU	1.96	19.7%	46.8%	42.7%	84.9%	2021-30		
F110	FCU	2.37	31.6%	46.7%	48.9%	82.2%	2021-30	1	PP499173
F148	FCU	2.37	32.9%	27.8%	15.0%	58.5%	2021-		
F086	FEQ	1.86	50.0%	47.9%	50.1%	78.3%	2021-26	1	PP499152
F101	FEQ	2.04	38.2%	57.5%	46.3%	78.5%	2021-30	1	PP499167
F102	FEQ	2.21	40.8%	56.1%	54.2%	84.1%	2021-30		
F108	FEQ	2.52	40.8%	54.1%	42.5%	78.0%	2021-30	1	PP499172
CS346	FFL	1.42	16.4%	6.9%	5.3%	54.0%	2022-4	1	PP499079
F029	FFL	1.72	17.1%	8.9%	24.9%	75.1%	2021-16	1	PP499098
F149	FGR	2.19	44.7%	2.4%	24.7%	64.7%	2021-	1	PP499194

CS171	FOX	1.94	27.9%	13.9%	-8.0%	50.1%	2022-44		
CS174	FOX	2.22	59.0%	33.1%	8.6%	68.1%	2022-46		
CS218	FOX	1.78	26.2%	10.8%	-6.9%	45.5%	2022-26		
CS358	FOX	2.01	50.8%	20.5%	43.2%	63.2%	2022-44		
CS359	FOX	2.16	37.7%	10.1%	41.2%	61.0%	2022-44		
CS459	FOX	1.95	45.9%	23.3%	26.3%	62.7%	2022-26		
CS466	FOX	1.48	14.8%	2.5%	-13.7%	47.7%	2022-26	1	PP499083
F150	FOX	2.45	59.2%	11.1%	47.2%	79.7%	2021-	1	PP499195
F152	FPR	1.71	14.5%	-9.7%	-6.7%	41.2%	2021-	1	PP499196
CS293	FRE	1.49	52.5%	13.1%	30.4%	70.4%	2022-18		
CS298	FRE	1.53	23.0%	14.6%	31.3%	68.6%	2022-32	1	PP499074
CS316	FRE	1.33	26.2%	4.9%	-18.6%	57.8%	2022-48	1	PP499075
CS319	FRE	1.23	14.8%	-2.9%	-26.9%	44.2%	2022-9	1	PP499076
CS327	FRE	1.27	31.1%	12.0%	16.4%	53.5%	2022-26	1	PP499077
CS341	FRE	1.46	19.7%	7.5%	5.3%	51.1%	2022-44	1	PP499078
CS457	FRE	2.04	37.7%	15.4%	15.7%	57.4%	2022-44	1	PP499081
F038	FRE	1.62	10.5%	14.3%	29.4%	82.2%	2021-18	1	PP499107
F042	FRE	0.83	9.2%	2.5%	29.5%	17.5%	2021-19	1	PP499110
F043	FRE	0.64	1.3%	-18.6%	-11.6%	30.8%	2021-19	1	PP499111
F045	FRE	0.58	1.3%	3.8%	2.2%	34.7%	2021-19		
F047	FRE	1.80	17.1%	16.4%	41.8%	65.1%	2021-21	1	PP499114
F048	FRE	1.71	30.3%	27.4%	52.2%	74.8%	2021-23	1	PP499115
F050	FRE	1.10	17.1%	17.6%	39.8%	64.4%	2021-23	1	PP499116
F083	FRE	1.46	19.7%	37.7%	29.8%	73.4%	2021-26	1	PP499149
F084	FRE	1.34	1.3%	30.0%	5.6%	72.6%	2021-26	1	PP499150
F087	FRE	1.86	5.3%	33.8%	17.2%	74.0%	2021-26	1	PP499153
F089	FRE	1.03	11.8%	35.4%	-2.6%	69.7%	2021-26	1	PP499155

F092	FRE	1.70	15.8%	41.0%	19.5%	75.8%	2021-27		
F103	FRE	1.65	13.2%	47.2%	28.7%	77.4%	2021-30	1	PP499168
F113	FRE	1.43	7.9%	31.3%	1.2%	73.1%	2021-30		
F116	FRE	1.97	11.8%	42.6%	9.9%	76.5%	2021-30	1	PP499178
F120	FRE	1.76	27.6%	35.7%	26.0%	76.0%	2021-30	1	PP499180
F121	FRE	1.81	17.1%	27.4%	6.6%	71.3%	2021-30		
F135	FRE	1.28	9.2%	-2.1%	-67.9%	11.9%	2021-33	1	PP499187
CS226	FSO	1.55	31.1%	10.6%	-24.3%	55.1%	2022-16		
CS227	FSO	1.69	23.0%	7.6%	-16.5%	53.7%	2022-20		
CS233S	FSO	1.48	16.4%	6.3%	-10.2%	65.1%	2022-26		
CS258	FSO	2.30	77.0%	54.9%	58.8%	87.7%	2022-9		
CS344	FSO	1.39	21.3%	5.3%	1.7%	39.4%	2022-32		
CS349	FSO	1.39	24.6%	9.9%	21.8%	59.0%	2022-9		
CS352	FSO	1.33	26.2%	15.5%	31.8%	53.7%	2022-12	1	PP499080
CS464	FSO	1.19	19.7%	-5.3%	-15.5%	36.3%	2022-21	1	PP499082
F015	FSO	1.06	13.2%	22.3%	40.4%	68.9%	2021-11	1	PP499087
F021	FSO	0.92	9.2%	14.6%	24.6%	59.4%	2021-13	1	PP499090
F023	FSO	1.56	14.5%	8.6%	19.4%	79.5%	2021-15	1	PP499092
F041	FSO	0.97	9.2%	10.8%	35.2%	23.3%	2021-19	1	PP499109
F044	FSO	0.61	5.3%	4.7%	13.4%	50.4%	2021-19	1	PP499112
F051	FSO	1.50	14.5%	26.8%	54.2%	75.6%	2021-23	1	PP499117
F057	FSO	2.54	2.6%	24.2%	56.9%	87.0%	2021-24	1	PP499123
F098	FSO	1.61	3.9%	39.9%	-1.6%	80.2%	2021-29	1	PP499162
F100	FSO	1.61	10.5%	37.0%	8.6%	67.8%	2021-29	1	PP499166
F111	FSO	1.65	7.9%	38.8%	18.4%	79.1%	2021-30	1	PP499174
F112	FSO	1.74	6.6%	39.9%	10.5%	77.4%	2021-30	1	PP499175
F114	FSO	1.74	7.9%	30.7%	10.8%	53.7%	2021-30	1	PP499176

F118	FSO	1.74	9.2%	36.6%	0.1%	72.7%	2021-30	1	PP499179
F065	FSP	4.00	100.0%	91.2%	96.4%	96.6%	2021-25	1	PP499130
F105	FSP	4.00	100.0%	100.0%	98.6%	99.6%	2021-30	1	PP499169
CS255	FTO	1.36	24.6%	16.2%	-14.0%	75.5%	2022-13		
CS289	FTO	1.63	16.4%	6.9%	-9.6%	49.7%	2022-21		
F007	FTO	1.19	11.8%	29.9%	23.0%	67.6%	2021-3	1	PP499145
F014	FTO	2.17	44.7%	34.7%	43.9%	67.0%	2021-11	1	PP499086
F019	FTO	1.42	6.6%	27.2%	36.1%	67.2%	2021-12	1	PP499088
F033	FTO	1.78	19.7%	5.0%	32.6%	79.1%	2021-16	1	PP499103
F035	FTO	1.76	14.5%	6.4%	24.4%	79.0%	2021-16	1	PP499105
F070	FTO	2.25	26.3%	10.3%	19.2%	78.5%	2021-25	1	PP499136
F115	FTO	1.82	42.1%	40.7%	21.5%	78.3%	2021-30	1	PP499177
F122	FTO	1.83	13.2%	32.9%	17.9%	73.9%	2021-30	1	PP499181
F140	FTO	1.77	13.2%	-4.3%	-33.7%	46.9%	2021-33	1	PP499191
F142	FTO	1.92	13.2%	6.4%	-15.8%	51.7%	2021-33	1	PP499192
F146	FTO	2.58	9.2%	9.0%	22.7%	69.4%	2021-33	1	PP499193
CS056	FTR	1.89	11.5%	2.4%	-12.8%	43.9%	2022-20	1	PP499059
CS362	FTR	1.96	23.0%	-8.6%	12.0%	50.3%	2022-6		
F010	FTR	1.78	13.2%	17.1%	20.3%	62.6%	2021-4	1	PP499084

[a]FAC, *Fusarium acuminatum*; FAV, *Fusarium avenaceum*; FCO, *Fusarium commune*; FCU, *Fusarium culmorum*; FEQ, *Fusarium equiseti*; FFL, *Fusarium flocciferum*; FGR, *Fusarium graminearum*; FOX, *Fusarium oxysporum*; FPR, *Fusarium proliferatum*; FRE, *Fusarium redolens*; FSO, *Fusarium solani*; FSP, *Fusarium sporotrichioides*; Fsp, unidentified species; FTO, *Fusarium torulosum*, FTR, *Fusarium tricinctum*; [b]Root rot disease severity (0-4 scale) at 21 days after seeding [42]; [c]Reduction on seedling emergence caused by corresponding isolate compared with non-inoculated control; [d]Reduction on plant height caused by corresponding isolate compared with non-inoculated control; [e]Reduction on shoot dry weight caused by corresponding isolate compared with non-inoculated control; [f]Reduction on root dry weight caused by corresponding isolate compared with non-inoculated control; [g]Isolated year and field number; [h]Isolates involved in phylogenetic tree construction: 1 represents involved in tree construction; [i]NCBI-assigned accession numbers.

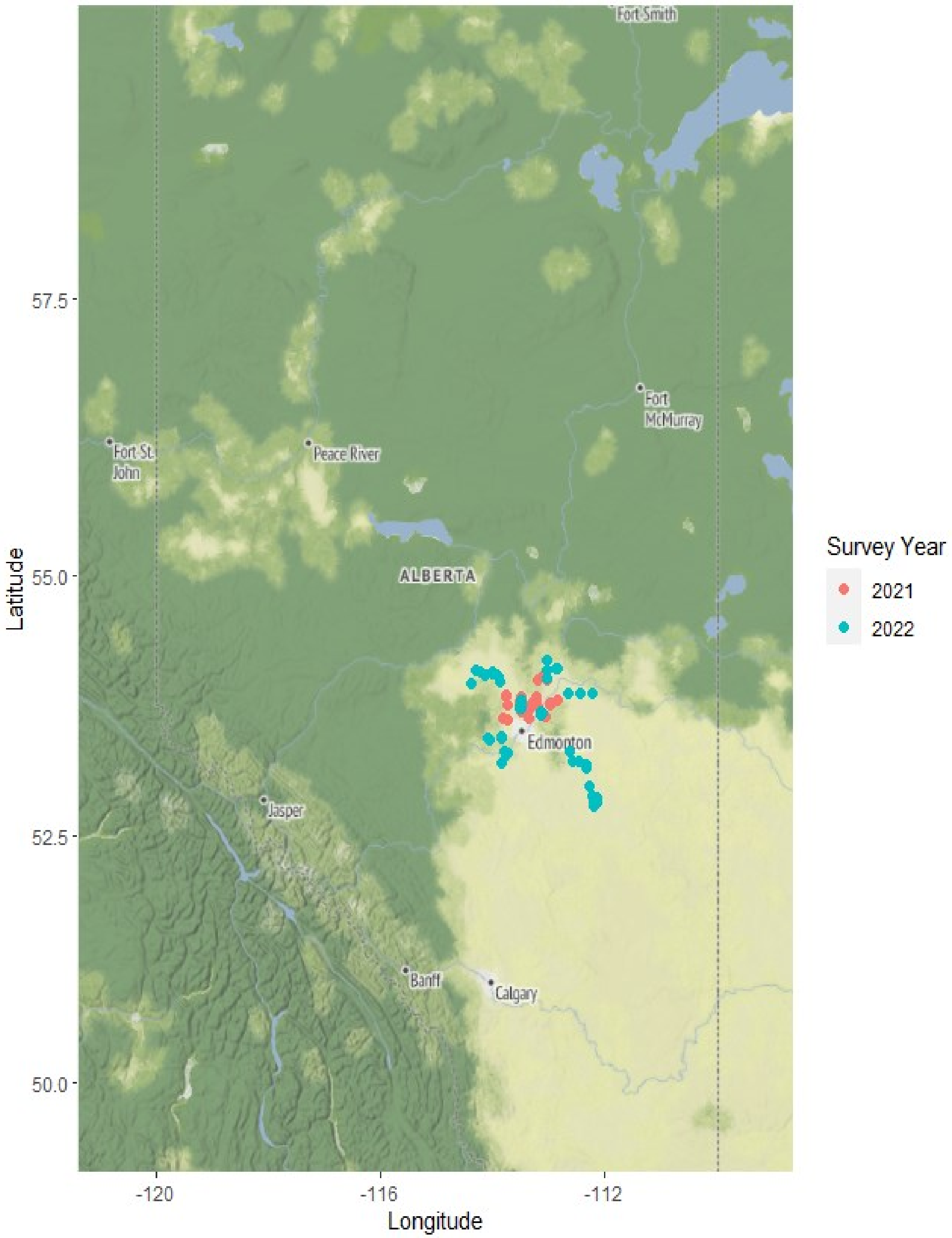

Supplementary Figure S1. Fields surveyed for the incidence of canola root rot in 2021 and 2022. Longitude and latitude indicate the position of surveyed fields on the maps. Fields visited in 2021 and 2022 are indicated in red and blue, respectively

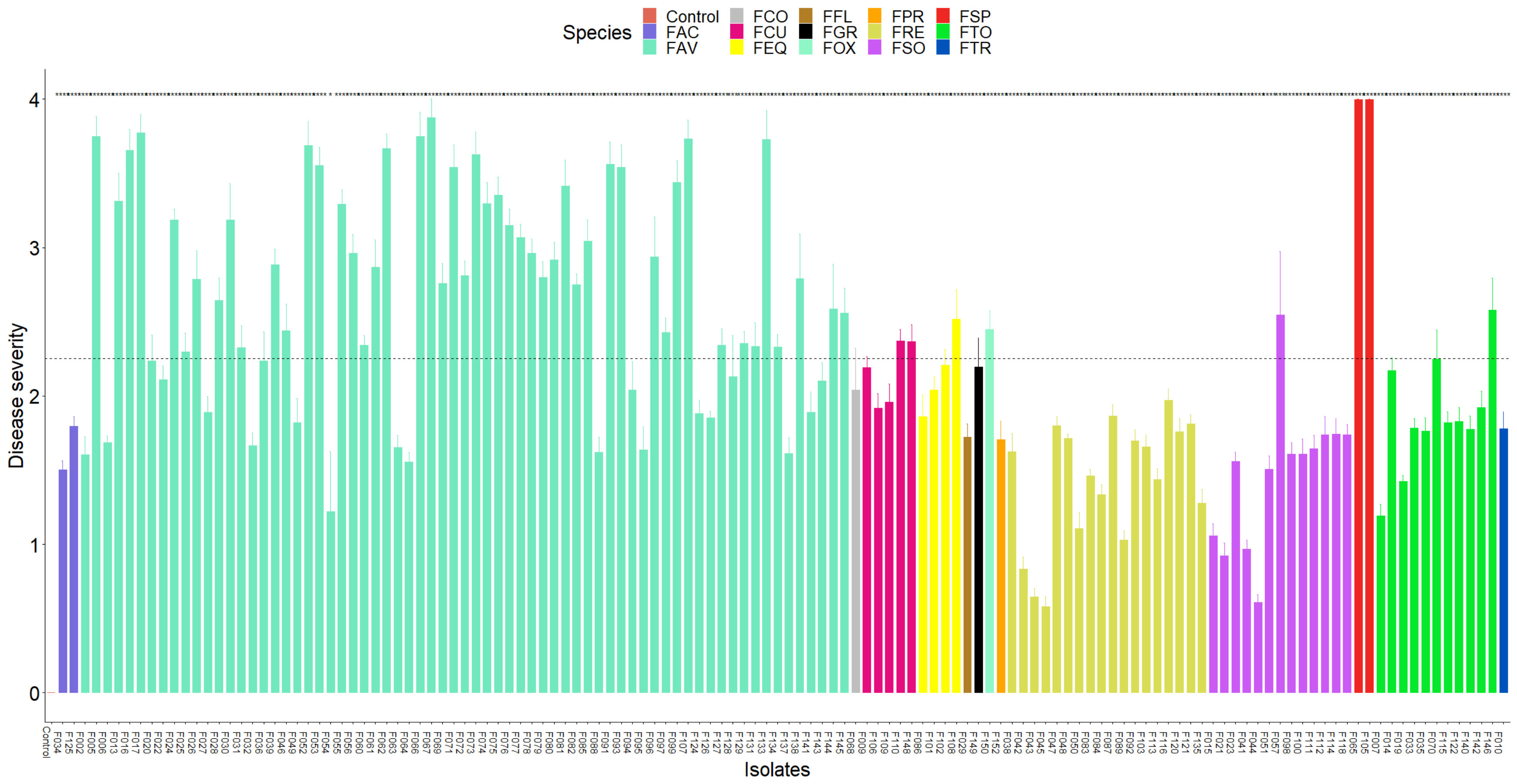

Supplementary Figure S2A. Impact of *Fusarium* isolates, identified from 2021, on root rot disease severity of canola cv. 'Westar' under greenhouse conditions. Species, species identity; Control, non-inoculated control; FAC, *Fusarium acuminatum*; FAV, *Fusarium avenaceum*; FCO, *Fusarium commune*; FCU, *Fusarium culmorum*; FEQ, *Fusarium equiseti*; FFL, *Fusarium flocciferum*; FGR, *Fusarium graminearum*; FOX, *Fusarium oxysporum*; FPR, *Fusarium proliferatum*; FRE, *Fusarium redolens*; FSO, *Fusarium solani*; FSP, *Fusarium sporotrichioides*; FTO, *Fusarium torulosum*; FTR, *Fusarium tricinctum*; ns, no significant difference between the

treatment and corresponding non-inoculated control based on a t-test; *, significant difference at $p < 0.05$; **, significant difference at $p < 0.01$; ***, significant difference at $p < 0.001$; and ****, significant difference at $p < 0.0001$. The dashed lines represent the overall mean for corresponding parameter.

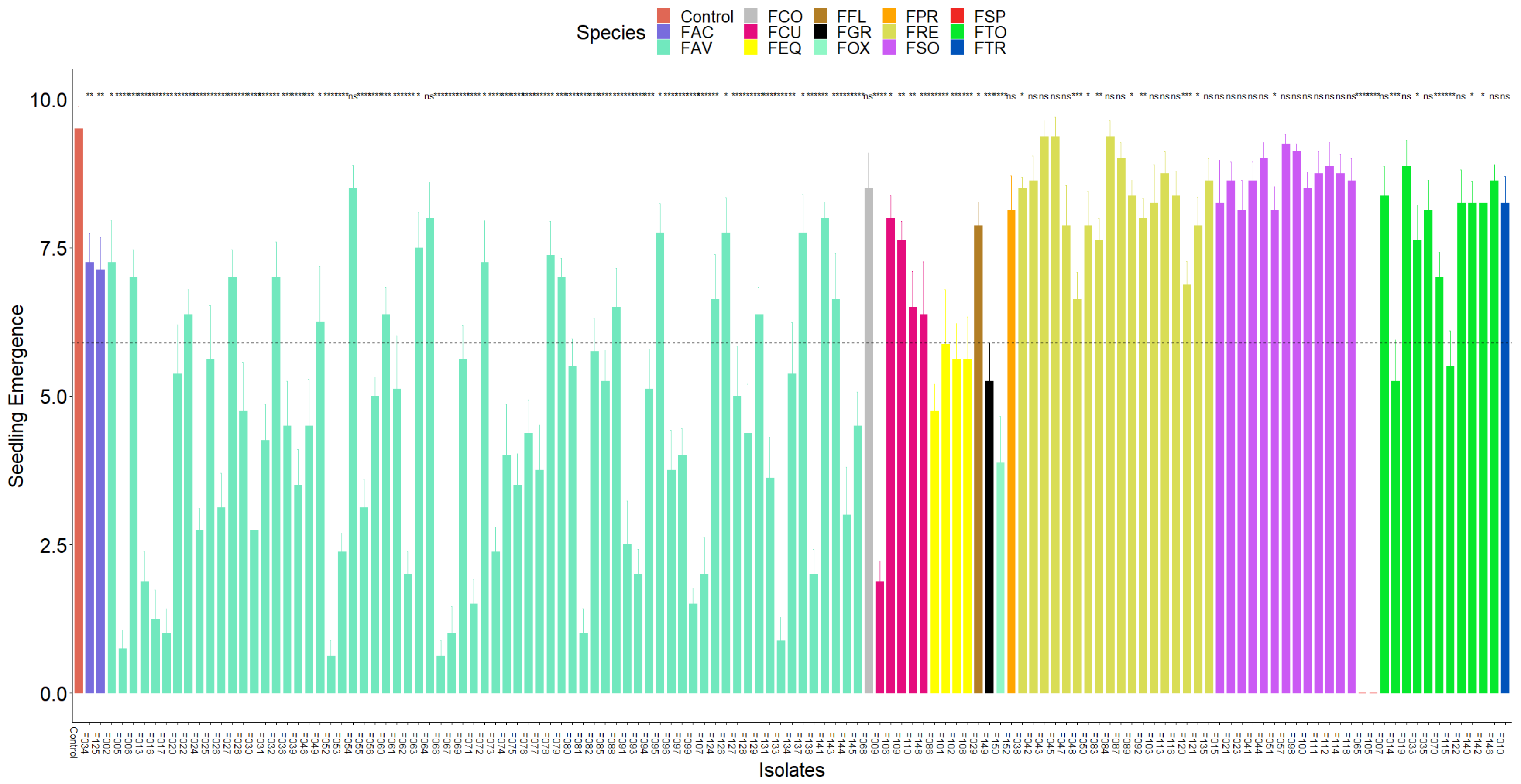

Supplementary Figure S2B. Impact of *Fusarium* isolates, identified from 2021, on seedling emergence of canola cv. 'Westar' under greenhouse conditions. Species, species identity; Control, non-inoculated control; FAC, *Fusarium acuminatum*; FAV, *Fusarium avenaceum*; FCO, *Fusarium commune*; FCU, *Fusarium culmorum*; FEQ, *Fusarium equiseti*; FFL, *Fusarium flocciferum*; FGR, *Fusarium graminearum*; FOX, *Fusarium oxysporum*; FPR, *Fusarium proliferatum*; FRE, *Fusarium redolens*; FSO, *Fusarium solani*; FSP, *Fusarium sporotrichioides*; FTO, *Fusarium torulosum*; FTR, *Fusarium tricinctum*; ns, no significant difference between the

treatment and corresponding non-inoculated control based on a t-test; *, significant difference at $p < 0.05$; **, significant difference at $p < 0.01$; ***, significant difference at $p < 0.001$; and ****, significant difference at $p < 0.0001$. The dashed lines represent the overall mean for corresponding parameter.

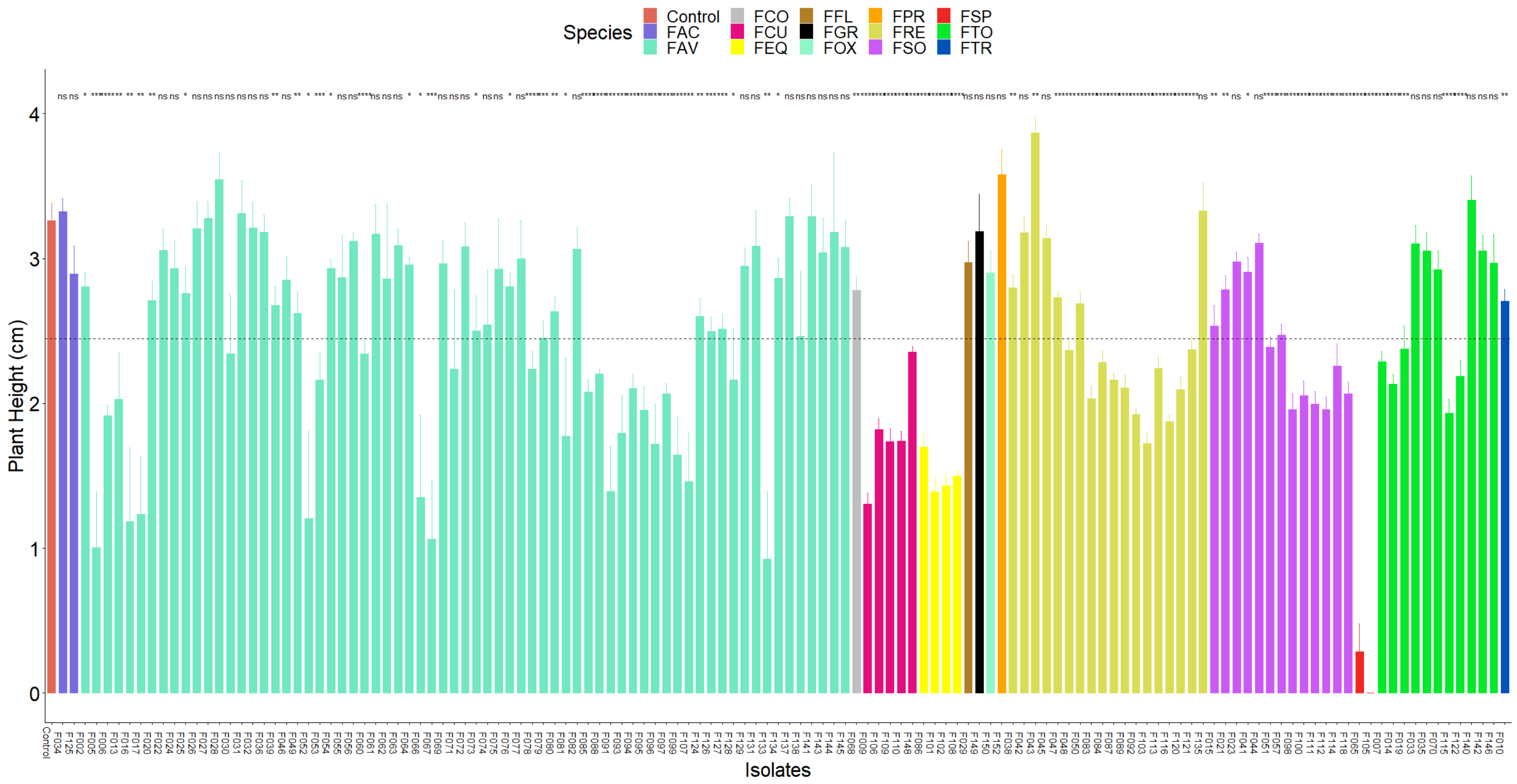

Supplementary Figure S2C. Impact of *Fusarium* isolates, identified from 2021, on plant height of canola cv. 'Westar' under greenhouse conditions. Species, species identity; Control, non-inoculated control; FAC, *Fusarium acuminatum*; FAV, *Fusarium avenaceum*; FCO, *Fusarium commune*; FCU, *Fusarium culmorum*; FEQ, *Fusarium equiseti*; FFL, *Fusarium flocciferum*; FGR, *Fusarium graminearum*; FOX, *Fusarium oxysporum*; FPR, *Fusarium proliferatum*; FRE, *Fusarium redolens*; FSO, *Fusarium solani*; FSP, *Fusarium sporotrichioides*; FTO, *Fusarium torulosum*; FTR, *Fusarium tricinctum*; ns, no significant difference between the treatment and

corresponding non-inoculated control based on a t-test; *, significant difference at $p < 0.05$; **, significant difference at $p < 0.01$; ***, significant difference at $p < 0.001$; and ****, significant difference at $p < 0.0001$. The dashed lines represent the overall mean for corresponding parameter.

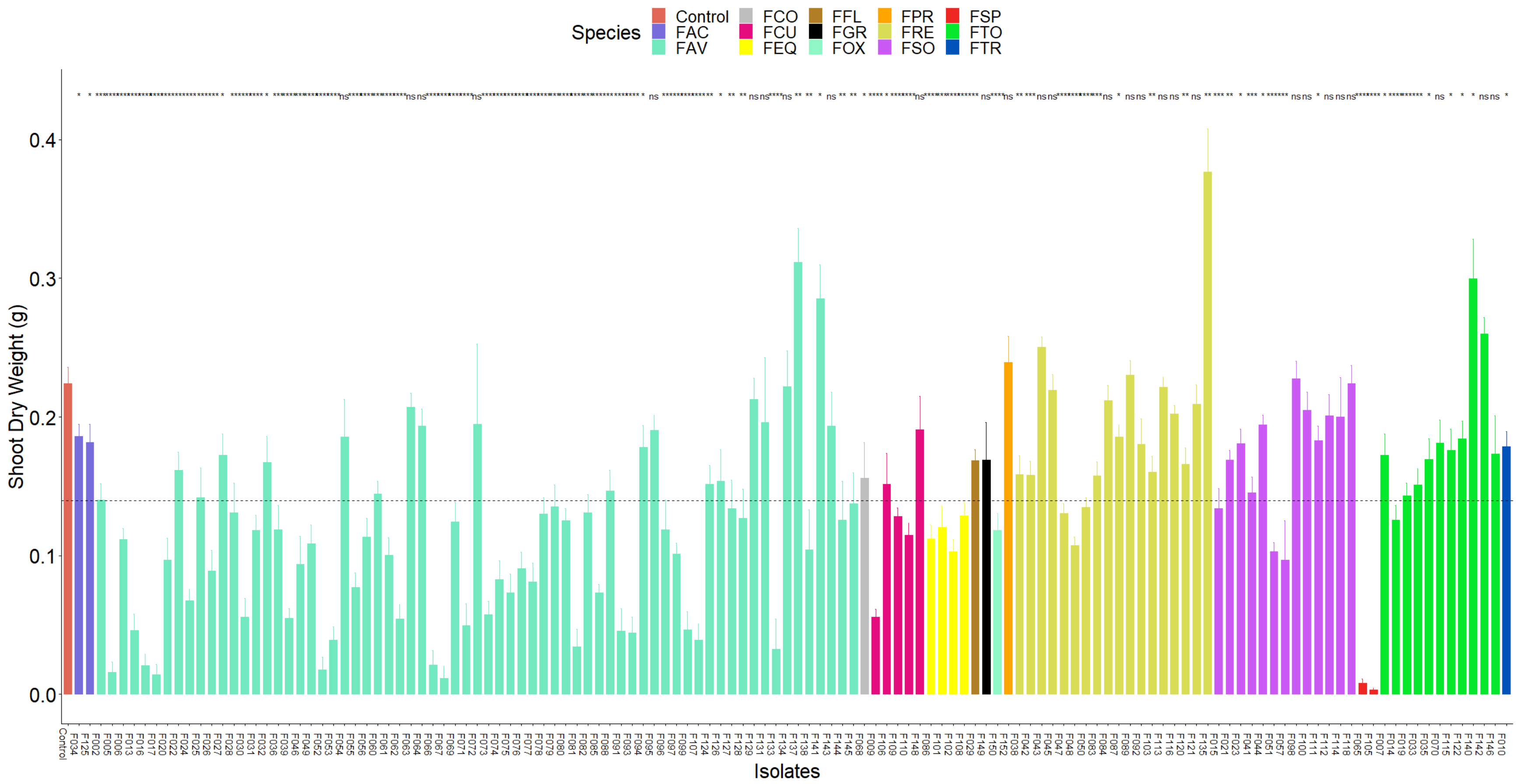

Supplementary Figure S2D. Impact of *Fusarium* isolates, identified from 2021, on shoot dry weight of canola cv. 'Westar' under greenhouse conditions. Species, species identity; Control, non-inoculated control; FAC, *Fusarium acuminatum*; FAV, *Fusarium avenaceum*; FCO, *Fusarium commune*; FCU, *Fusarium culmorum*; FEQ, *Fusarium equiseti*; FFL, *Fusarium flocciferum*; FGR, *Fusarium graminearum*; FOX, *Fusarium oxysporum*; FPR, *Fusarium proliferatum*; FRE, *Fusarium redolens*; FSO, *Fusarium solani*; FSP, *Fusarium sporotrichioides*; FTO, *Fusarium torulosum*; FTR, *Fusarium tricinctum*; ns, no significant difference between the

treatment and corresponding non-inoculated control based on a t-test; *, significant difference at $p < 0.05$; **, significant difference at $p < 0.01$; ***, significant difference at $p < 0.001$; and ****, significant difference at $p < 0.0001$. The dashed lines represent the overall mean for corresponding parameter.

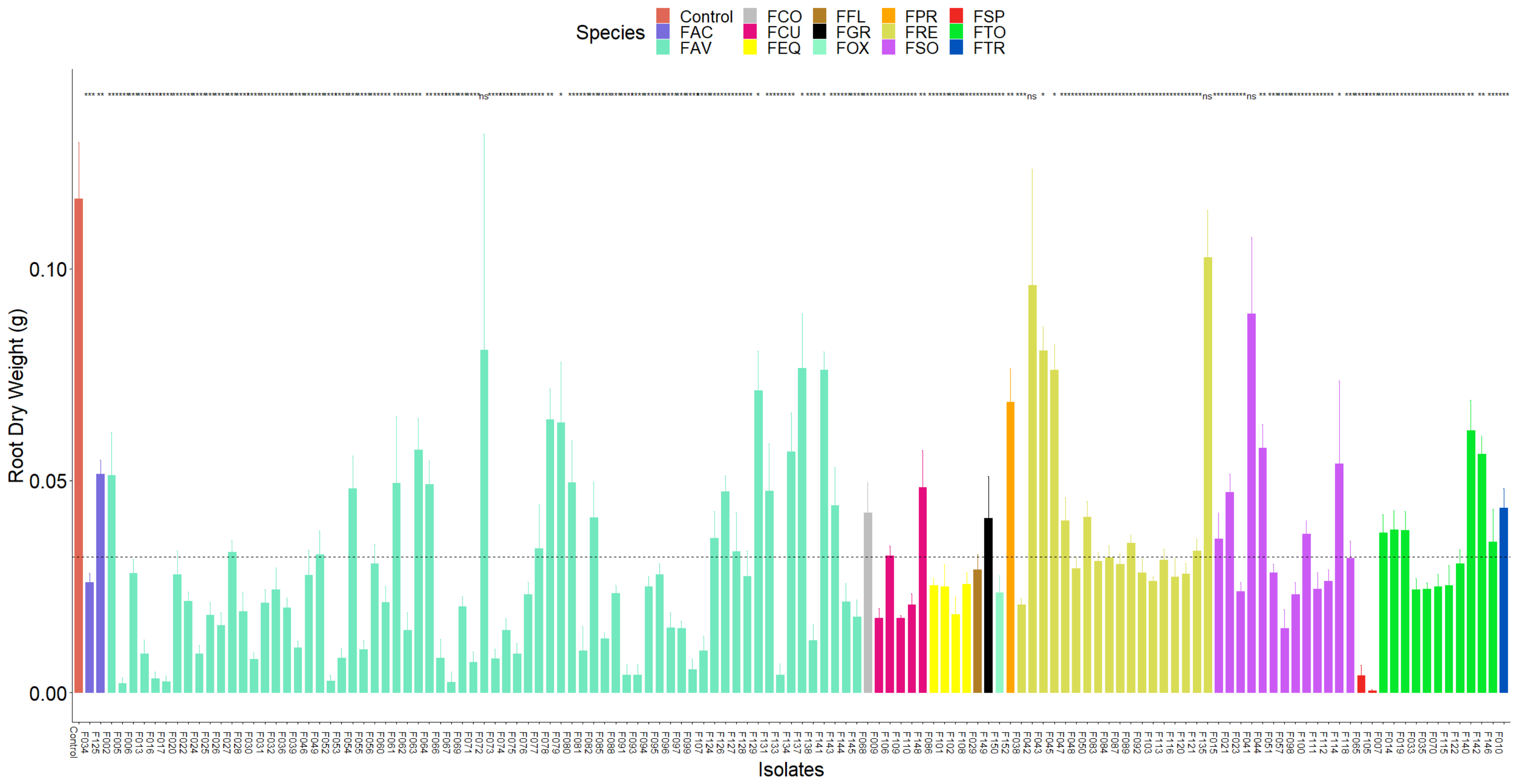

Supplementary Figure S 2E. Impact of *Fusarium* isolates, identified from 2021, on root dry weight of canola cv. 'Westar' under greenhouse conditions. Species, species identity; Control, non-inoculated control; FAC, *Fusarium acuminatum*; FAV, *Fusarium avenaceum*; FCO, *Fusarium commune*; FCU, *Fusarium culmorum*; FEQ, *Fusarium equiseti*; FFL, *Fusarium flocciferum*; FGR,*Fusarium graminearum*; FOX, *Fusarium oxysporum*; FPR, *Fusarium proliferatum*; FRE, *Fusarium redolens*; FSO , *Fusarium solani*;FSP, *Fusarium sporotrichioides*; FTO, *Fusarium torulosum*; FTR, *Fusarium tricinctum*; ns, no significant difference between the

treatment and corresponding non-inoculated control based on a t-test; *, significant difference at $p < 0.05$; **, significant difference at $p < 0.01$; ***, significant difference at $p < 0.001$; and ****, significant difference at $p < 0.0001$. The dashed lines represent the overall mean for corresponding parameter.

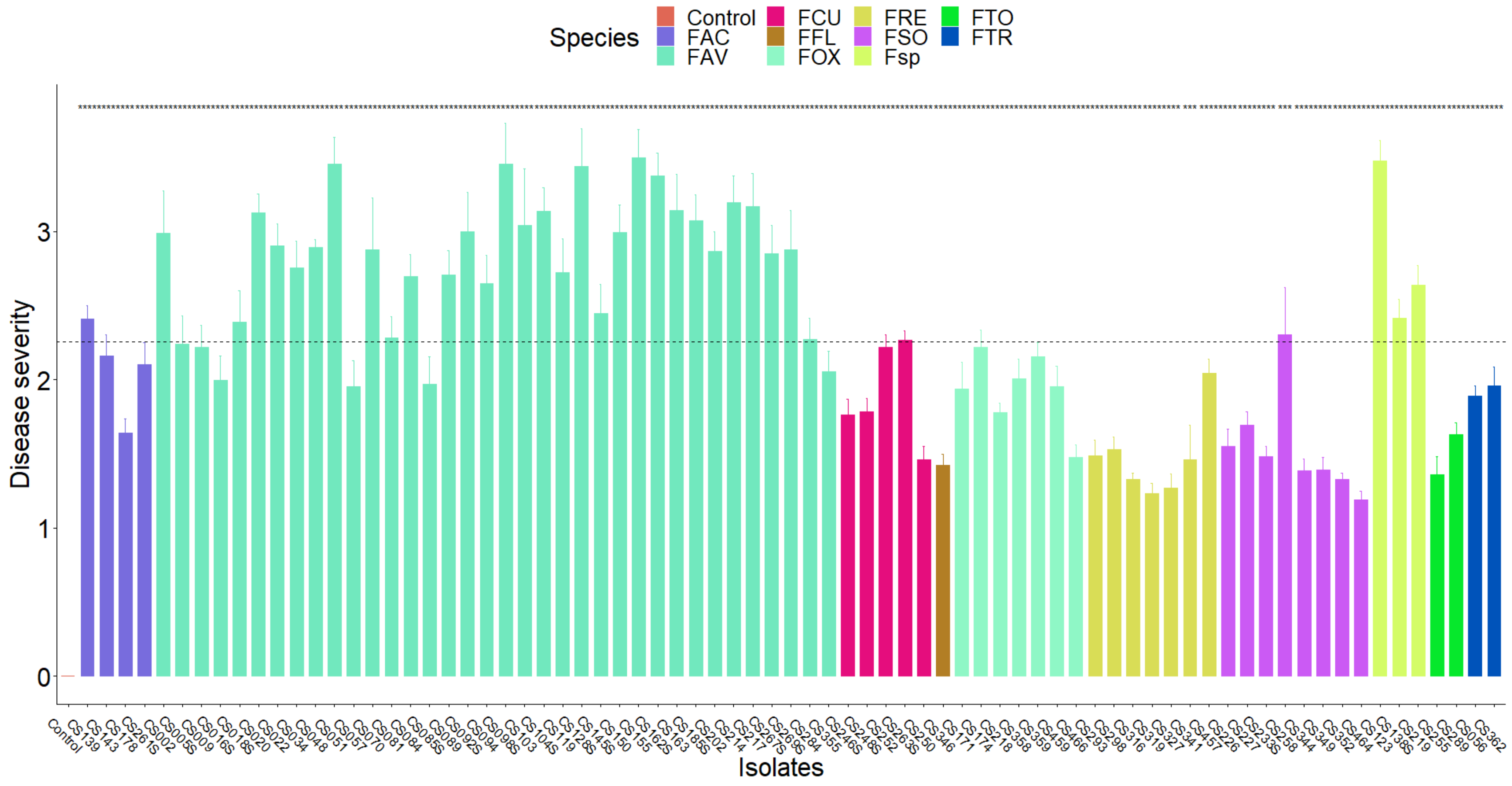

Supplementary Figure S3A. Impact of *Fusarium* isolates, identified from 2022, on root rot disease severity of canola cv. 'Westar' under greenhouse conditions. Species, species identity; Control, non-inoculated control; FAC, *Fusarium acuminatum*; FAV, *Fusarium avenaceum*; FCU, *Fusarium culmorum*; FFL, *Fusarium flocciferum*; FOX, *Fusarium oxysporum*; FRE, *Fusarium redolens*; FSO, *Fusarium solani*; Fsp, unidentified *Fusarium* sp.; FTO, *Fusarium torulosum*; FTR, *Fusarium tricinctum*; ns, no significant difference between the treatment and corresponding non-inoculated control based on a t-test; *, significant difference at $p < 0.05$; **, significant

difference at $p < 0.01$; ***, significant difference at $p < 0.001$; and ****, significant difference at $p < 0.0001$. The dashed lines represent the overall mean for corresponding parameter.

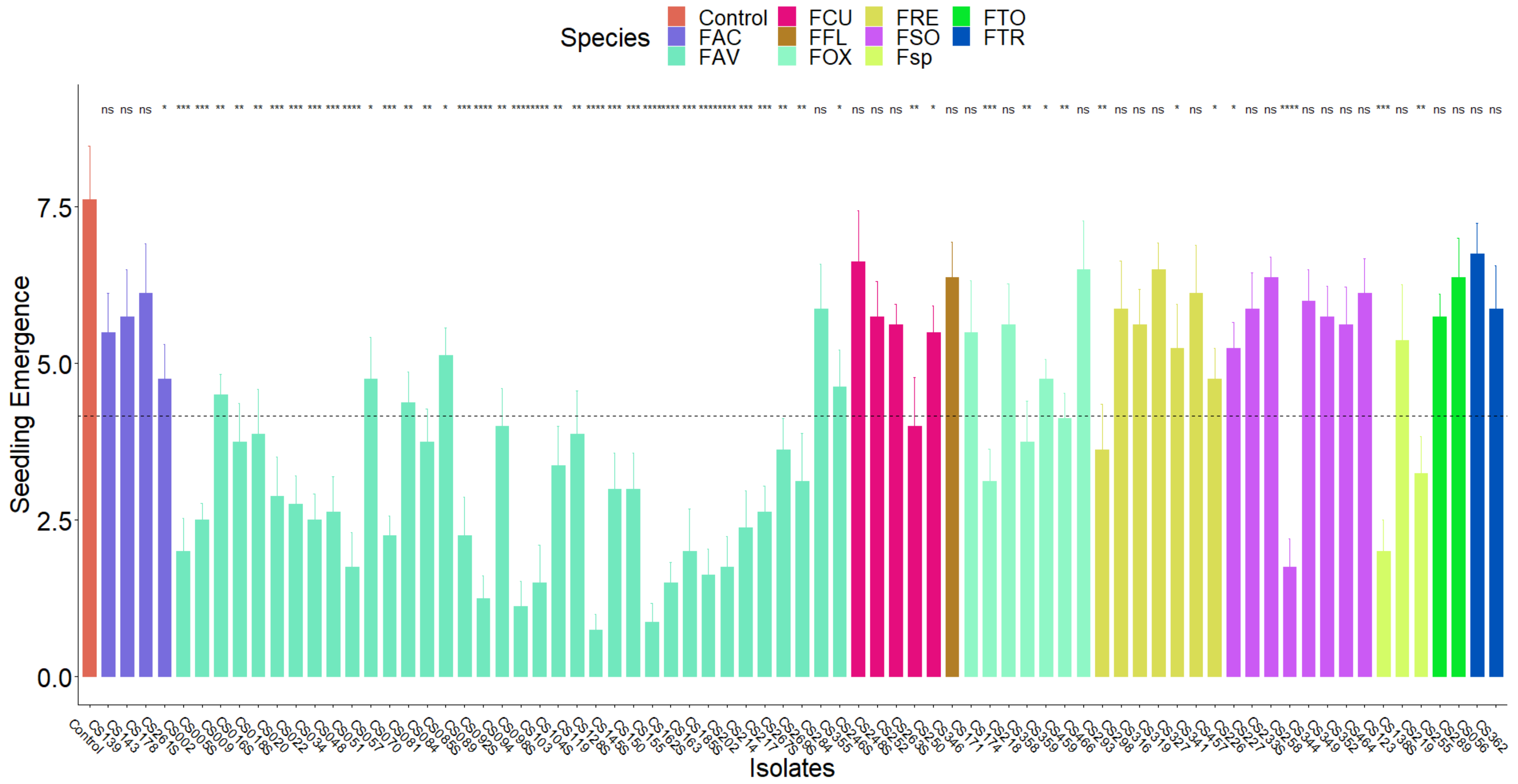

Supplementary Figure S3B. Impact of *Fusarium* isolates, identified from 2022, on seedling emergence of canola cv. 'Westar' under greenhouse conditions. Species, species identity; Control, non-inoculated control; FAC, *Fusarium acuminatum*; FAV, *Fusarium avenaceum*; FCU, *Fusarium culmorum*; FFL, *Fusarium flocciferum*; FOX, *Fusarium oxysporum*; FRE, *Fusarium redolens*; FSO, *Fusarium solani*; Fsp, unidentified *Fusarium* sp.; FTO, *Fusarium torulosum*; FTR, *Fusarium tricinctum*; ns, no significant difference between the treatment and corresponding non-inoculated control based on a t-test; *, significant difference at $p < 0.05$; **, significant

difference at $p < 0.01$; ***, significant difference at $p < 0.001$; and ****, significant difference at $p < 0.0001$. The dashed lines represent the overall mean for corresponding parameter.

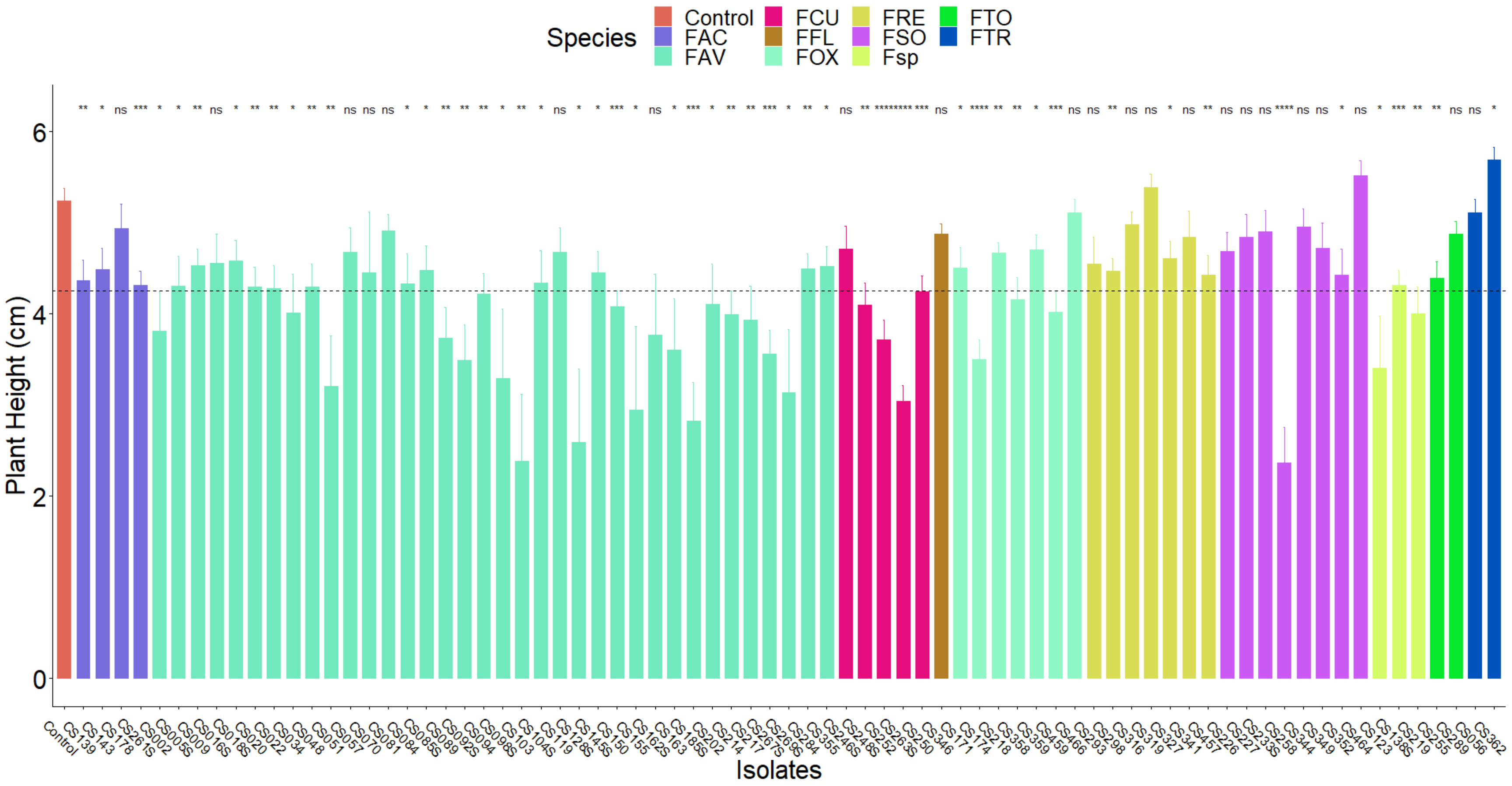

Supplementary Figure S3C. Impact of *Fusarium* isolates, identified from 2022, on plant height of canola cv. 'Westar' under greenhouse conditions. Species, species identity; Control, non-inoculated control; FAC, *Fusarium acuminatum*; FAV, *Fusarium avenaceum*; FCU, *Fusarium culmorum*; FFL, *Fusarium flocciferum*; FOX, *Fusarium oxysporum*; FRE, *Fusarium redolens*; FSO, *Fusarium solani*; Fsp, unidentified *Fusarium* sp.; FTO, *Fusarium torulosum*; FTR, *Fusarium tricinctum*; ns, no significant difference between the treatment and corresponding non-inoculated control based on a t-test; *, significant difference at $p < 0.05$; **, significant difference at $p < 0.01$;

, significant difference at $p < 0.001$; and *, significant difference at $p < 0.0001$. The dashed lines represent the overall mean for corresponding parameter.

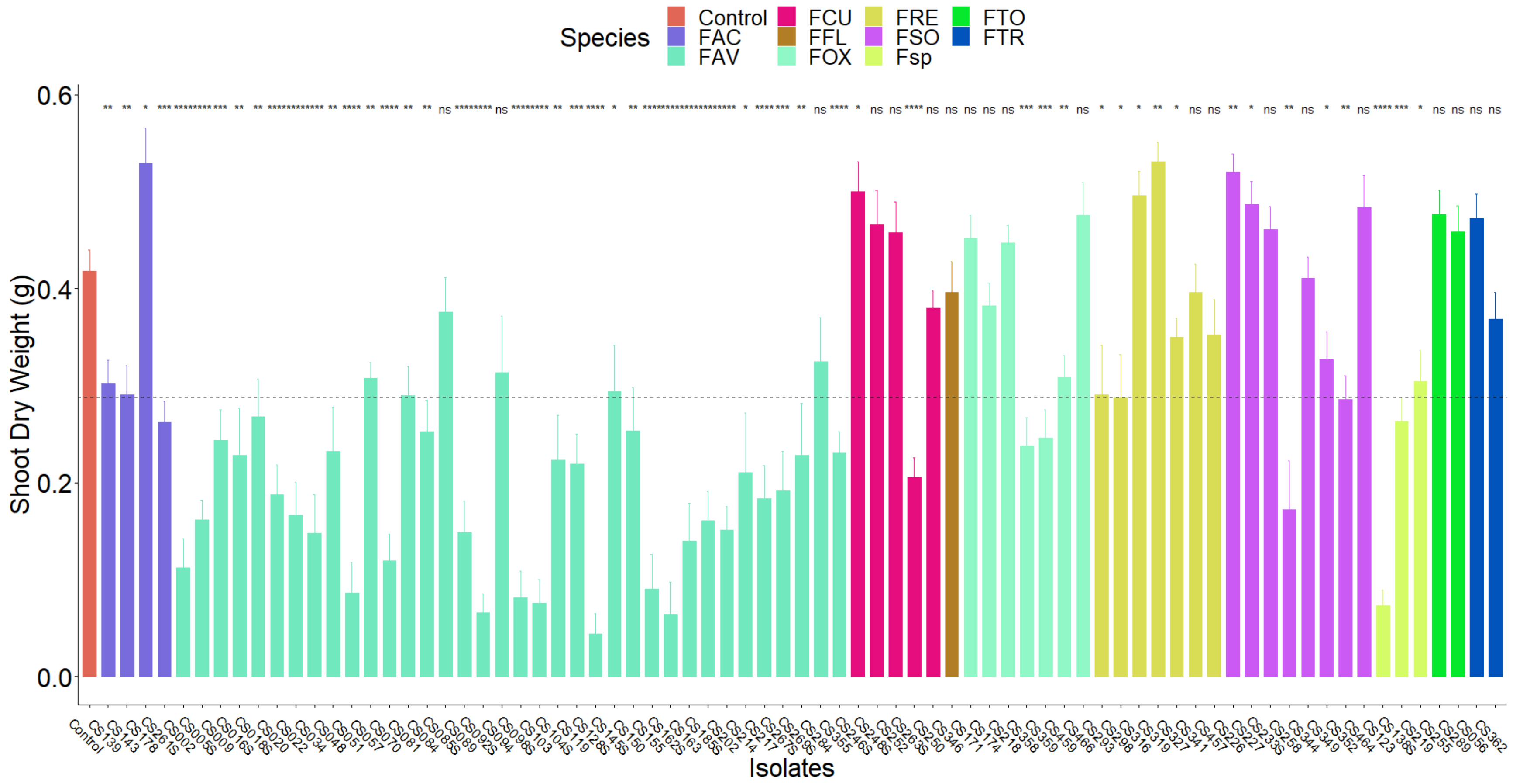

Supplementary Figure S3D. Impact of *Fusarium* isolates, identified from 2022, on shoot dry weight of canola cv. 'Westar' under greenhouse conditions. Species, species identity; Control, non-inoculated control; FAC, *Fusarium acuminatum*; FAV, *Fusarium avenaceum*; FCU, *Fusarium culmorum*; FFL, *Fusarium flocciferum*; FOX, *Fusarium oxysporum*; FRE, *Fusarium redolens*; FSO, *Fusarium solani*; Fsp, unidentified *Fusarium* sp.; FTO, *Fusarium torulosum*; FTR, *Fusarium tricinctum*; ns, no significant difference between the treatment and corresponding non-inoculated control based on a t-test; *, significant difference at $p < 0.05$; **, significant

difference at $p < 0.01$; ***, significant difference at $p < 0.001$; and ****, significant difference at $p < 0.0001$. The dashed lines represent the overall mean for corresponding parameter.

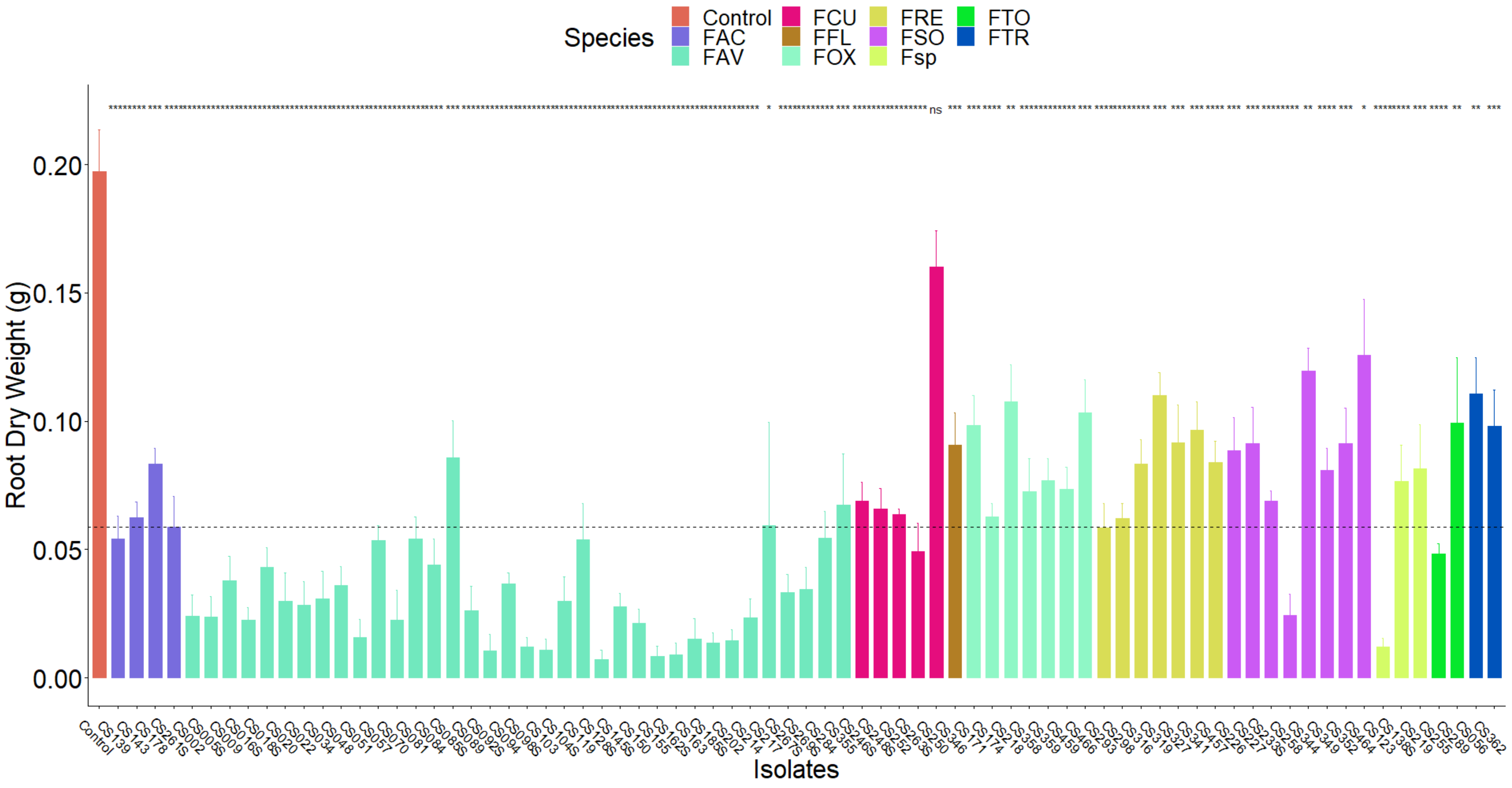

Supplementary Figure S3E. Impact of *Fusarium* isolates, identified from 2022, on root dry weight of canola cv. 'Westar' under greenhouse conditions. Species, species identity; Control, non-inoculated control; FAC, *Fusarium acuminatum*; FAV, *Fusarium avenaceum*; FCU, *Fusarium culmorum*; FFL, *Fusarium flocciferum*; FOX, *Fusarium oxysporum*; FRE, *Fusarium redolens*; FSO, *Fusarium solani*; Fsp, unidentified *Fusarium* sp.; FTO, *Fusarium torulosum*; FTR, *Fusarium tricinctum*; ns, no significant difference between the treatment and corresponding non-inoculated control based on a t-test; *, significant difference at $p < 0.05$; **, significant

difference at $p < 0.01$; ***, significant difference at $p < 0.001$; and ****, significant difference at $p < 0.0001$. The dashed lines represent the overall mean for corresponding parameter.

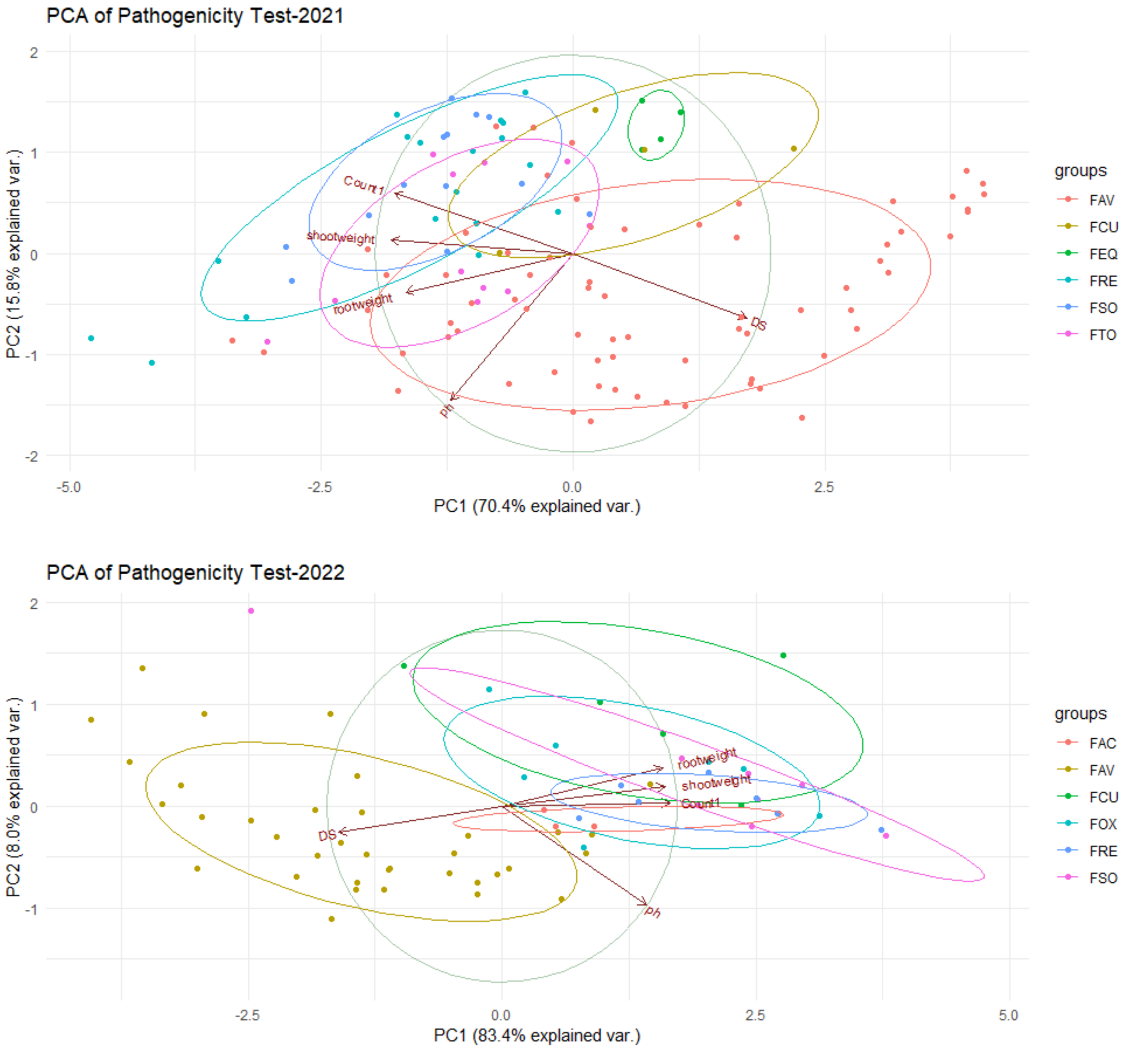

Supplementary Figure S4. Principal component analysis based on root rot disease severity (DS) and seedling emergence (Count1), plant height (ph), shoot dry weight (shootweight), and root dry weight (rootweight) of canola seedlings after inoculation with the isolates from the common identified six *Fusarium* species in 2021 (top) and 2021 (bottom). groups, species identity; FAV, *Fusarium avenaceum*; FCU, *Fusarium culmorum*; FRE, *Fusarium redolens*; FTO, *Fusarium torulosum*; FSO, *Fusarium solani*; FEQ, *Fusarium equiseti*; FOX, *Fusarium oxysporum*; FAC, *Fusarium acuminatum*.

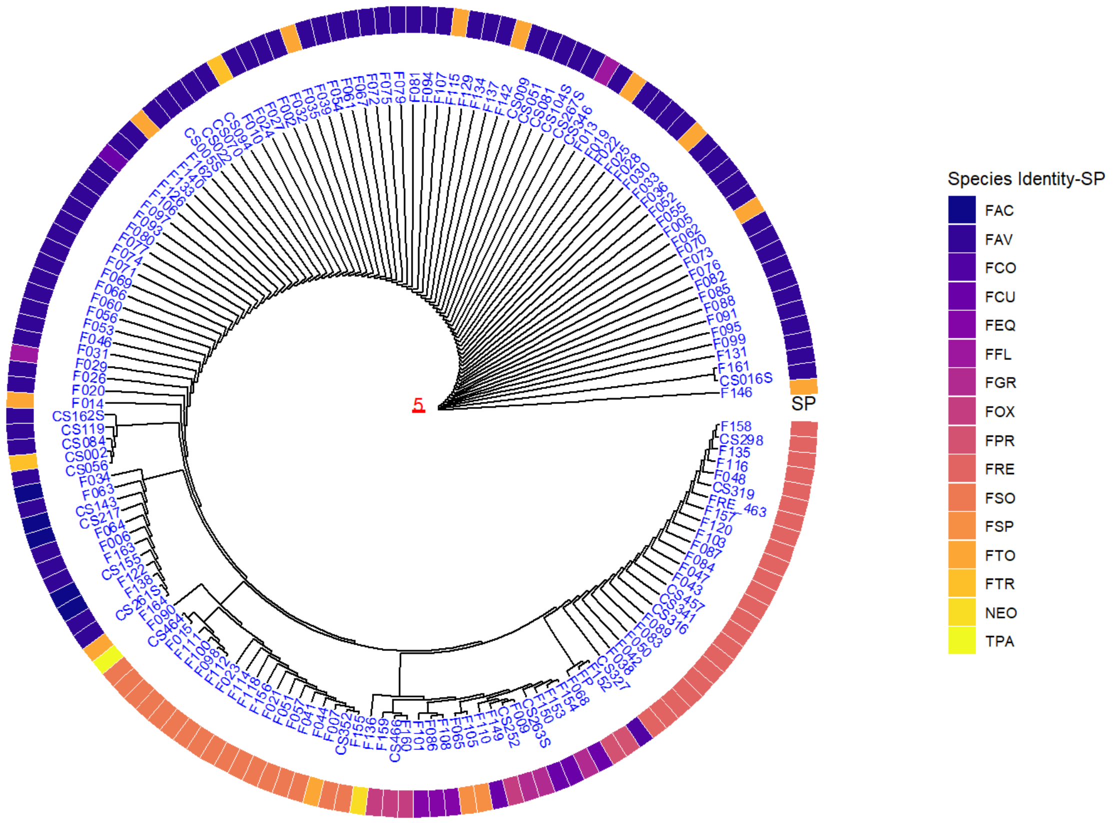

Supplementary Figure S5. Maximum parsimony tree based on the internal transcribed spacer (ITS) sequence of 157 fungal isolates, including 112 isolates recovered from canola in 2021 (F002-F146), 31 isolates recovered from canola in 2022 (CS002-CS466), 13 reference isolates from a laboratory culture collection (FP, F153-F164), and sequences from one *Fusarium redolens* isolate FRE_463 retrieved from GenBank, National Center for Biotechnology Information (NCBI). SP, species identity; FAC, *Fusarium acuminatum*; FAV, *Fusarium avenaceum*; FCO, *Fusarium commune*; FCU, *Fusarium culmorum*; FEQ, *Fusarium equiseti*; FFL, *Fusarium flocciferum*; FGR, *Fusarium graminearum*; FOX, *Fusarium oxysporum*; FPR, *Fusarium proliferatum*; FRE, *Fusarium redolens*; FSO, *Fusarium solani*; FSP, *Fusarium sporotrichioides*; FTO, *Fusarium torulosum*; FTR, *Fusarium tricinctum*; NEO, *Neonectria* sp.; and TPA, *Trichoderma paraviridescens*. DS, disease severity on rated on a 0 to 4 scale (Hwang et al., 1994), where: 0 = healthy roots and 4 = tap root severely girdled, brown lesions on >75% of the tap root with limited lateral roots. Growth Losses, reductions in seedling emergence (CR), plant height (HR), shoot dry weight (SR), and root dry weight (RR) following inoculation with each fungal isolate and relative to the corresponding non-inoculated control.

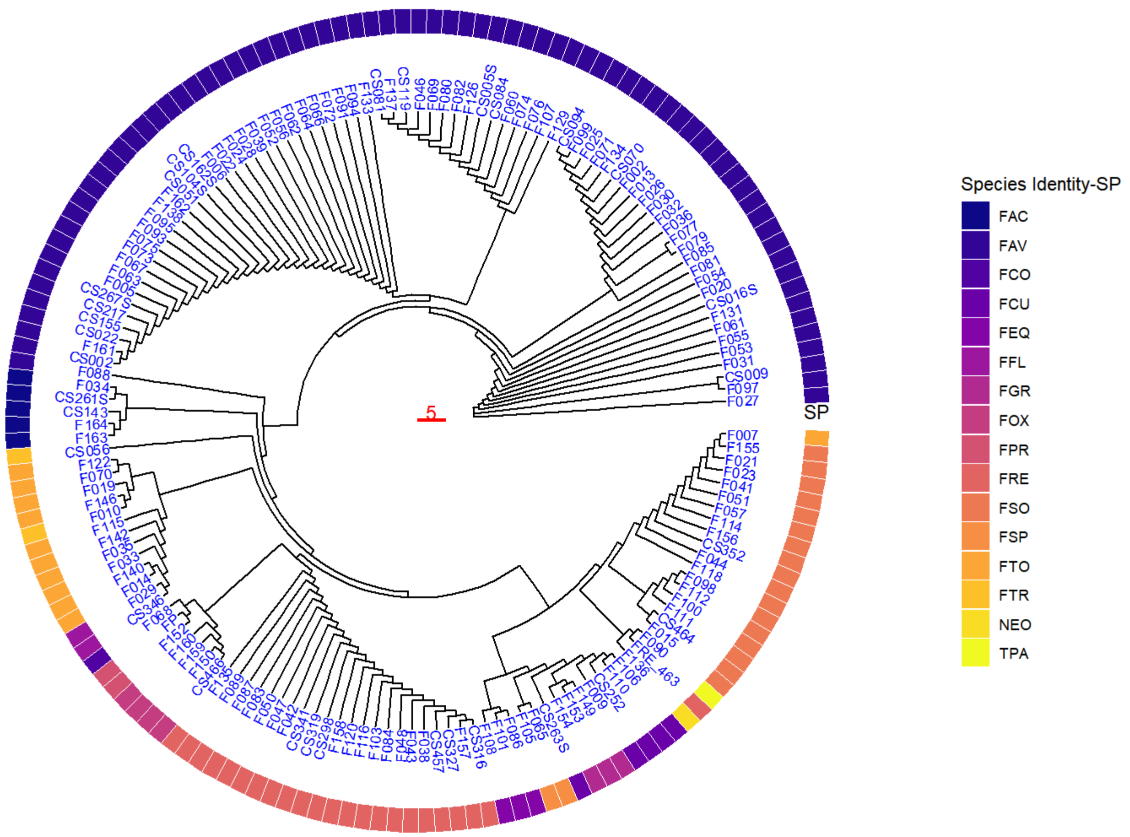

Supplementary Figure S6. Maximum parsimony tree based on the elongation factor (EF1-α) sequence of 157 fungal isolates, including 112 isolates recovered from canola in 2021 (F002-F146), 31 isolates recovered from canola in 2022 (CS002-CS466), 13 reference isolates from a laboratory culture collection (FP, F153-F164), and sequences from one *Fusarium redolens* isolate FRE_463 retrieved from GenBank, National Center for Biotechnology Information (NCBI). SP, species identity; FAC, *Fusarium acuminatum*; FAV, *Fusarium avenaceum*; FCO, *Fusarium commune*; FCU, *Fusarium culmorum*; FEQ, *Fusarium equiseti*; FFL, *Fusarium flocciferum*; FGR, *Fusarium graminearum*; FOX, *Fusarium oxysporum*; FPR, *Fusarium proliferatum*; FRE, *Fusarium redolens*; FSO, *Fusarium solani*; FSP, *Fusarium sporotrichioides*; FTO, *Fusarium torulosum*; FTR, *Fusarium tricinctum*; NEO, *Neonectria* sp.; and TPA, *Trichoderma paraviridescens*. DS, disease severity on rated on a 0 to 4 scale (Hwang et al., 1994), where: 0 = healthy roots and 4 = tap root severely girdled, brown lesions on >75% of the tap root with limited lateral roots. Growth Losses, reductions in seedling emergence (CR), plant height (HR), shoot dry weight (SR), and root dry weight (RR) following inoculation with each fungal isolate and relative to the corresponding non-inoculated control.
